# ﻿An annotated checklist of the psyllids (Hemiptera, Psylloidea) of Norfolk Island with keys to species, new records, and descriptions of two new endemic species

**DOI:** 10.3897/zookeys.1238.124535

**Published:** 2025-05-19

**Authors:** Francesco Martoni, James M. H. Tweed, Mark J. Blacket, Diana M. Percy

**Affiliations:** 1 Agriculture Victoria Research, AgriBio Centre, 5 Ring Road, Bundoora, VIC 3083, Australia Agriculture Victoria Research, AgriBio Centre Bundoora Australia; 2 School of the Environment, Centre for Biodiversity and Conservation Science, University Dr, Goddard Building, The University of Queensland, St Lucia, Brisbane, QLD 4072, Australia The University of Queensland Brisbane Australia; 3 Department of Botany and Biodiversity Research Centre, 6270 University Boulevard, University of British Columbia, Vancouver, V6T 1Z4, Canada University of British Columbia Vancouver Canada

**Keywords:** *Acizzia*, *Alyxia*, Australia, *Dodonaea*, endemism, New Zealand, *Pseudophacopteron*, Southwest Pacific, taxonomy

## Abstract

Norfolk Island is a small, isolated archipelago in the Pacific Ocean, 1400 km east of the Australian mainland. The history of human colonisation and land use on the island has resulted in a substantial reduction in the extent and quality of indigenous habitat. A quarantine survey of Norfolk Island in 2012–2014 provided the first records of psyllid species, reporting six taxa from the island. Additional collection records are provided that increase the number to 14 species, of which nine are regarded as adventive, four as native of which two are endemic, and one whose additional distribution is unknown. Two species are formally described here and are the first psyllid species to be described from Norfolk Island. These new species, *Pseudophacopteronaewagriini* Percy & Martoni, **sp. nov.** (Aphalaridae) and *Acizziaaliceae* Percy & Martoni, **sp. nov.** (Psyllidae) are both considered endemic to Norfolk Island and are associated with native plants, the endemic *Alyxiagynopogon* (Apocynaceae) and the native *Dodonaeaviscosa* (Sapindaceae), respectively. In addition to an updated checklist, identification keys to adults and immatures of the psyllids found on Norfolk Island and DNA barcodes for all species are provided. Both new species have had complete mitochondrial genomes sequenced in a previous study and here a full annotation of the mitochondrial genome of *Acizziaaliceae* Percy & Martoni, **sp. nov.** is supplied. Lastly, the barcode data was analysed in a maximum likelihood constraint framework with previous genome data to investigate the phylogenetic origins of the Norfolk Island taxa.

## ﻿Introduction

The Norfolk Island group is a small, isolated archipelago located in the southwest Pacific Ocean (29.0408°S, 167.9547°E). Norfolk Island itself is the largest island in the group (35.6 km^2^) reaching an elevation of 319 m a.s.l., while the smaller Phillip (1.95 km^2^) and Nepean (0.09 km^2^) islands are located 5.8 km and 0.8 km to the south of Norfolk, respectively. New Caledonia and New Zealand are the nearest major landmasses and are located approximately 700 km to the north and south, respectively, while Lord Howe Island and mainland Australia lie approximately 900 km and 1,400 km to the west, respectively. The island group, referred to here as Norfolk Island for simplicity, has been heavily impacted by humans, particularly following colonisation by Europeans in 1788, with much of the indigenous vegetation cleared to make way for agriculture (Levin and Kark 2023). Only relatively small areas of indigenous vegetation remain, most of which occur within the Mt Pitt section of Norfolk Island National Park (Invasive Species Council 2021).

The superfamily Psylloidea (Hemiptera: Sternorrhyncha) is composed of almost 4000 described species worldwide (Ouvrard 2020), across seven different families (Burckhardt et al. 2021), including a few economically important species known to vector plant pathogens (Jagoueix et al. 1994; Liefting et al. 2009; Munyaneza et al. 2010). Like many invertebrate taxa from Norfolk Island, the psyllid fauna is poorly known. An extensive quarantine survey between 2012 and 2014 produced the first records of psyllids from Norfolk Island, as well as the first records for many other taxa (Walker et al. 2015; Maynard et al. 2018). Prior to this, no psyllids had been reported from Norfolk Island (Smithers 1998).

A total of six psyllid species belonging to three different families were recorded: three Aphalaridae, one Carsidaridae, and two Triozidae (Maynard et al. 2018). At the time, the Norfolk Island psyllid fauna included introductions of taxa native to the two countries with closest economic and political ties, Australia (three species) and New Zealand (two species), while a single species appeared to be native to the area. In recent years, increasing efforts have been focused on exploring the psylloid diversity of the Southwest Pacific region (Martoni et al. 2016, 2024; Martoni and Brown 2018). The geographical location of Norfolk Island puts it in a central position between New Zealand, Australia and New Caledonia, giving the archipelago an important role in evaluating biogeographic distributions as well as anthropogenic-mediated dispersal and introduction of species due to high movement of people and produce. For these reasons, during a 2022 survey conducted to target plant pests and pathogens (Martoni et al. 2023), psyllids were targeted for collection.

In this study, we provide an updated and annotated checklist for the psyllids of Norfolk Island. We record eight previously unreported taxa and provide formal descriptions of two new species representing the first endemic psyllids described from Norfolk Island. We also provide identification keys to adults and immatures, and list DNA sequence resources (previous and newly generated) for these taxa. Lastly, we investigate the phylogenetic origins of the Norfolk Island psyllids using a maximum likelihood backbone constraint analysis.

## ﻿Materials and methods

### ﻿Sampling and field collections

Fresh specimens were collected by FM, MJB and JMHT during field trips in March and October 2022, February and March 2023, and October and November 2023. Collections were made by beating foliage over a beating tray. Numerous tree and shrub species were examined as potential psyllid hosts, based on the presence of psyllids from these host genera in other regions. These included species of *Acacia*, *Alyxia*, *Casuarina*, *Celtis*, *Dodonaea*, *Eucalyptus*, *Ficus*, *Leucaena*, *Melicope*, *Myoporum*, *Nestegis*, *Pisonia*, *Pittosporum*, *Planchonella*, and *Zanthoxylum*. Insect specimens were preserved in high grade ethanol (> 80%) for further analysis. Type material and additional material examined is deposited in the Australian National Insect Collection (**ANIC**) in Canberra, in the Victorian Agricultural Insect Collection (**VAIC**) in Bundoora, and in the Naturhistorisches Museum of Basel (**NHMB**), Switzerland. Additional material examined by DMP, originally collected during the Norfolk Island Quarantine Survey 2012–2014 (Maynard et al. 2018), is preserved at the University of British Columbia (**DMPC**).

### ﻿Specimen preparation, measurements, drawings, and photographs

Microscope slide preparation, following the work of Taylor et al. (2016), was performed on 20 adult specimens, ten individuals (5 males and 5 females) of *Pseudophacopteronaewagriini* Percy & Martoni, sp. nov. and ten individuals (5 males and 5 females) of *Acizziaaliceae* Percy & Martoni, sp. nov. Additionally, three immature specimens of *Acizziaaliceae* Percy & Martoni, sp. nov. and four immature specimens of *Pseudophacopteronaewagriini* Percy & Martoni, sp. nov. were mounted on two separate slides.

**Figure 1.**
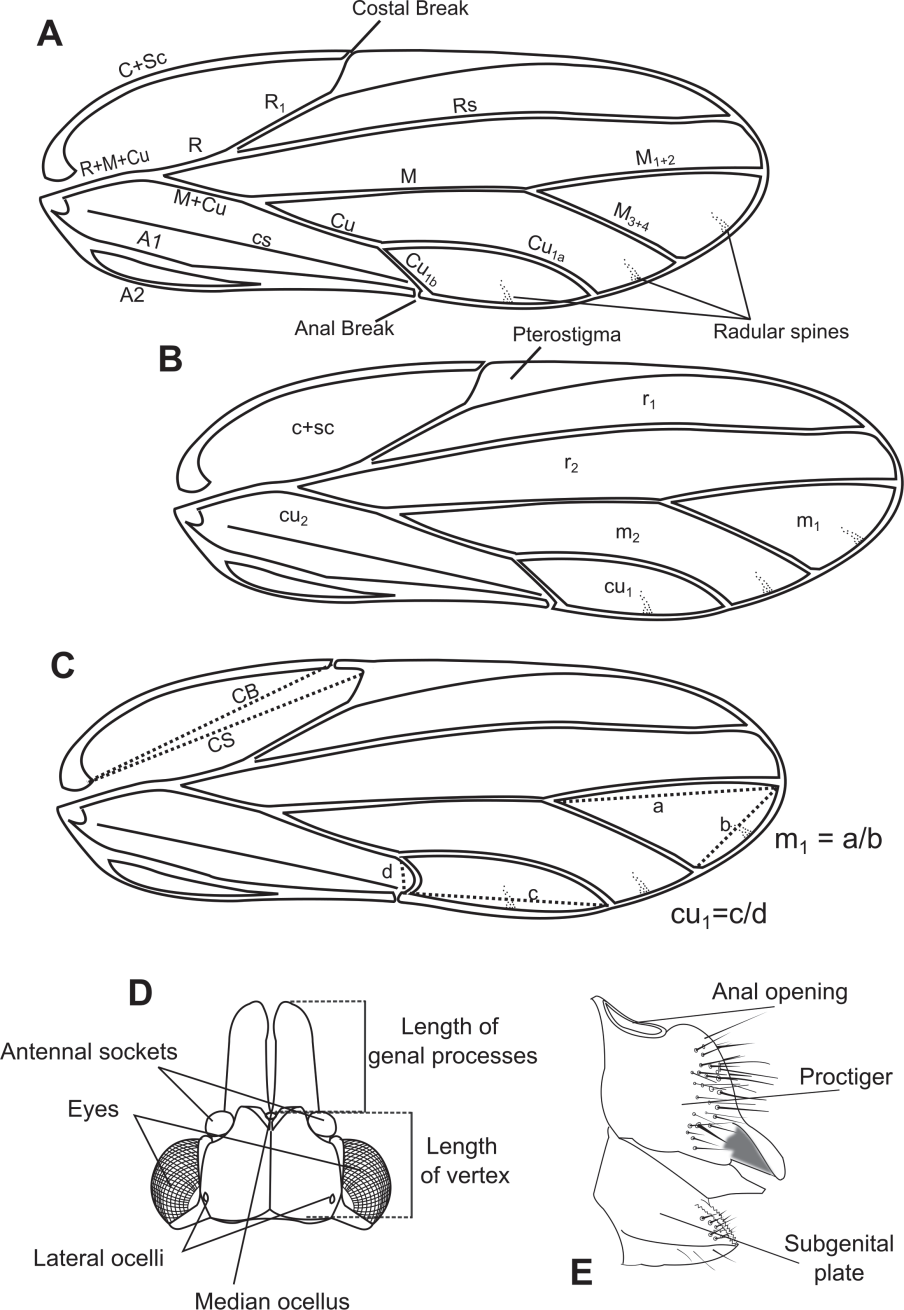
Morphological terminology **A** fore wing veins **B** cells **C** cell values and measurements **D** head, including measurements **E** female terminalia.

General morphology of adult characters follows that presented in the works of Taylor et al. (2016), Martoni and Blacket (2021), and Martoni et al. (2024), and it has been summarized in Fig. 1 for the purpose of the adult key presented here. Immature characters used in the key reference Taylor (1962, 1985, 1990), White and Hodkinson (1985), and Muddiman et al. (1992). Genus-specific measurements and ratios follow the work of Martoni and Armstrong (2019) for *Acizzia*, and Malenovský and Burckhardt (2009) and Malenovský et al. (2015) for *Pseudophacopteron*. High-resolution automontage photographs of adults were obtained using the Leica Application Suite software (version 4.5.0), from 5–20 stacked images obtained using a Leica stereo microscope M205C with a DFC450 camera (Leica Camera, Wetzlar, Germany). Additionally, a Hitachi TM3030Plus Tabletop Scanning electron microscope was used for close-up details (Hitachi, Tokyo, Japan). For the examination and high-resolution photographs of microscope slides, a Leica DM6 B microscope was used with a Leica DMC4500 camera (Leica Camera, Wetzlar, Germany) with additional images taken with a Zeiss Axioscope A1 microscope with Zeiss Axiocam 305 camera and stacked using HeliconFocus (v. 8.2.18). Measurements were obtained using the Leica ‘Segment Line Tool’ option.

High resolution photos were then collated into plates using the GNU Image Manipulation Program (GIMP) version.2.10.6. The line drawings were made using the software Inkscape v.1.2.2.

### ﻿Molecular analysis

DNA was non-destructively extracted from a total of 33 single specimens using the protocol of Martoni et al. (2020). A fragment of the subunit I of the *cytochrome oxidase* gene (COI) barcode region (Hebert et al. 2003) of ~ 570 bp was targeted using the primers Psy-COI-F3 (5’-ACAATTGTTACWGCWCAYGC-3’; Martoni et al. 2020) and HCO2198 (5’-TAAACTTCAGGGTGACCAAAAAATCA-3’; Folmer et al. 1994). The polymerase chain reaction (PCR) was performed using the MyFi kit (Bioline Meridian Biosciences, Cincinnati, USA) following the manufacturer’s instructions and the following cycle: initial denaturation at 95 °C for 5 min, followed by 40 cycles of 30 s at 94 °C, 30 s at 50 °C and 1 min at 72 °C, and a final elongation of 7 min at 72 °C. PCR products were Sanger sequenced in both directions commercially (Macrogen, Seoul, Korea). The electropherograms were manually examined and checked for pseudogenes and stop codons using the software MEGA X (Kumar et al. 2018). Forward and reverse sequences were combined in MEGA X and each sequence was blasted against the online databases GenBank and BOLD to assess similarities to other taxa. The sequences obtained were uploaded on the NCBI GenBank database, with accession numbers OR558292–OR558323, PQ999102 (Table 1).

**Table 1.** Norfolk Island specimens used for molecular analysis in this study and in Percy et al. (2018). The table includes information on the species recorded, including family, GenBank accession numbers for different genes, and collection dates. Accession numbers in bold were generated in this study.

Species	Family	Acc. Number	Gene	Coll. date
*Blastopsyllaoccidentalis*	Aphalaridae	**OR558304, OR558305**	COI	Oct-2022
		MG988657	COI	Dec-2012
		MG988950	cytB	Dec-2012
*Cardiaspinafiscella*	Aphalaridae	**OR558322, OR558323**	COI	Oct-2022
*Cryptoneossatriangula*	Aphalaridae	**OR558292**	COI	Mar-2022
*Glycaspisgranulata*	Aphalaridae	**OR558310, OR558311**	COI	Oct-2022
*Pseudophacopteronaewagriini* Percy & Martoni, sp. nov.	Aphalaridae	**OR558312–OR558315**	COI	Oct-2022
		MG988814	COI	Jul-2013
		MG988815	COI	Oct-2013
		MG989134	cytB	Oct-2013
		MG989135	cytB	Jul-2013
		MG989234	mitogenome	Jul-2013
*Mesohomotomahibisci*	Carsidaridae	**OR558306, OR558307**	COI	Oct-2022
		KY294175	COI	Dec-2012
		KY294659	cytB	Dec-2012
*Acizziaacaciaebaileyanae*	Psyllidae	**OR558293–OR558296**	COI	Feb-2023
		MG988623	COI	Jul-2013
		MG988894	cytB	Jul-2013
*Acizziahakeae*	Psyllidae	**PQ999102**	COI	Nov-2024
*Acizzia* sp. A	Psyllidae	**OR558303**	COI	Oct-2022
*Acizzia* sp. B	Psyllidae	**OR558297–OR558300**	COI	Feb-2023
*Acizziaaliceae* Percy & Martoni, sp. nov.	Psyllidae	**OR558301, OR558302; OR558308, OR558309**	COI COI	Feb-2023; Oct-2022
		MG988625	COI	Jul-2013
		MG988895	cytB	Jul-2013
		**PQ754209**	mitogenome	Jul-2013
*Heteropsyllacubana*	Psyllidae	**OR558320, OR558321**	COI	Oct-2022
*Bactericeracockerelli*	Triozidae	**OR558318, OR558319**	COI	Mar-2022
*Powelliavitreoradiata*	Triozidae	**OR558316, OR558317**	COI	Oct-2022
		KY294138	COI	Dec-2012
		KY294622	cytB	Dec-2012

Complete mitochondrial genomes for the two endemic species described here were generated in a previous study (Percy et al. 2018). For this study, the mitochondrial genome of *Acizziaaliceae* Percy & Martoni, sp. nov. has been annotated using Geneious R8 v8.1.9 (Biomatters Ltd., Kearse et al. 2012) and submitted to GenBank with accession number PQ754209.

To place the Norfolk Island species within the Psylloidea superfamily phylogeny presented in Percy et al. (2018), a maximum likelihood (ML) constraint analysis was run with RAxML v. 8.2.12 (Stamatakis 2014) on the CIPRES Science Gateway (Miller et al. 2010). The constraint analysis allows the placement of short DNA sequences within the broader phylogenetic framework with improved resolution. The constraint tree used was the total evidence tree obtained from the mitogenome data in Percy et al. (2018). The constraint tree option allows the user to specify an incomplete multifurcating constraint tree for the RaxML search. Initially, multifurcations are resolved randomly and the additional taxa are added using a maximum parsimony criterion to compute a comprehensive (containing all taxa) bifurcating tree (Stamatakis 2014). This tree is then further optimized under ML criteria respecting the given constraints with the added taxa unconstrained (i.e., can be placed in any part of the tree). Data partitions were specified for codon position and RNA regions, and ML search criteria employed model GTRCAT, 1000 rapid bootstraps, and Gamma optimisation of tree space.

### ﻿Threat classification

The threat status of species endemic to Norfolk Island was assessed as per the IUCN (2012) criteria. Occurrence records were compiled from collected specimens as well as confirmed identifications from iNaturalist (https://www.inaturalist.org/), and observational data provided by colleagues in the field. Extent of occurrence (EOO) for each species was calculated by generating convex hulls with the minimum bounding geometry function of QGIS. Area of occupancy (AOO) was calculated based on a 4 km^2^ grid overlain on Norfolk Island.

## ﻿Results

### ﻿Key to the psyllids of Norfolk Island (adults)

**Table:** 

1	Fore wing with R+M+Cu stem trifurcating into veins R, M and Cu (Fig. 2A, B) (Triozidae)	**2**
–	Fore wing with R+M+Cu stem bifurcating into veins R and M+Cu (Fig. 2C) (other families)	**3**
2	Fore wing apex bluntly acute, with vein Rs long, reaching margin of wing closer to wing apex (distance between apices of veins Rs and M_1+2_ subequal or less than distance between M_1+2_ and M_3+4_), bifurcation of vein M into M_1+2_ and M_3+4_ anterior to line connecting apices of veins Rs and Cu_1a_, shape of cell cu_1_ higher, with cu_1_ value < 2 (Fig. 2A). On *Pittosporum* spp. (Pittosporaceae)	***Powelliavitreoradiata***
–	Fore wing apex acute, with vein Rs shorter (distance between apices of veins Rs and M_1+2_ much greater than distance between M_1+2_ and M_3+4_), bifurcation of vein M into M_1+2_ and M_3+4_ at or posterior to line connecting apices of veins Rs and Cu_1a_, shape of cell cu_1_ lower, with cu_1_ value 2 or greater (Fig. 2B). On *Solanum* and *Capsicum* spp. (Solanaceae)	***Bactericeracockerelli***
3	Fore wing with veins Rs and M_1+2_ either connected by a cross vein (Fig. 2C) or meeting at a contact point (Fig. 3A)	**4**
–	Fore wing with veins Rs and M_1+2_ not connected or contacting (Fig. 3B, C)	**5**
4	Fore wing with no markings on membrane, apex bluntly acute, cell cu_1_ narrower than m_1_, costal break absent (Fig. 2C). On *Hibiscustiliaceus* (Malvaceae)	***Mesohomotomahibisci***
–	Fore wing with dark markings on membrane around the base and posterior margin, apex broadly rounded, cell cu_1_ wider than m_1_, costal break present (Fig. 3A). On *Alyxiagynopogon* (Apocynaceae)	***Pseudophacopteronaewagriini* Percy & Martoni, sp. nov.**
5	Fore wing with vein R longer than M+Cu (Fig. 3B). On Fabaceae and Sapindaceae (*Acacia*, *Leucaena*, *Dodonaea*)	**6 (Psyllidae)**
–	Fore wing with vein R shorter than M+Cu (Fig. 3C). On Myrtaceae (mostly *Eucalyptus*)	**11 (Aphalaridae)**
6	Fore wing with broad, short, somewhat triangular pterostigma (~ 0.3 × wing length), shorter Rs vein (~ 0.5 × fore wing length), low cell cu_1_ with value higher than 2 (height of cell cu_1_ ~ 0.18 × wing width) (Fig. 3D). Male terminalia with paramere deeply bifid, the two parts divided almost to the base, male proctiger without distinct posterior lobe. On *Leucaena*	***Heteropsyllacubana***
–	Fore wing with narrower and longer pterostigma (length 0.44–0.48 × wing length), longer Rs vein (length > 0.6 × fore wing length), high cell cu_1_ with values reaching maximum 1.5 (height of cell cu_1_ 0.27–0.33 × wing width) (Fig. 4G). Male terminalia with paramere not divided, male proctiger with distinct posterior lobe. On *Acacia* or *Dodonaea*	**7 (*Acizzia*)**
7	Fore wing membrane with distinct spotted or banded markings, often creating Y shapes at the apical margins of cells (Fig. 4A, C, E, F). On *Acacia*	**8**
–	Fore wing membrane without distinct markings of spots or bands, either clear or yellowish (Fig. 4G). On *Dodonaeaviscosa*	***Acizziaaliceae* Percy & Martoni, sp. nov.**
8	Larger species (total length ~ 3 mm, and fore wing length ~ 2 mm). Fore wing membrane with darker patches in the central part of the wing and around Cs (Fig. 4A). Host plant unknown	***Acizzia* sp. “A**”
–	Smaller species (total length ~ 2 mm, and fore wing length usually < 1.7 mm). Fore wing with spots more scattered and not forming large patches (Fig. 4C, E, F). On *Acacia*, *Hakea* or *Grevillea*	**9**
9	Fore wing with vein M strongly arcuate, also other veins more curved, particularly M_1+2_ and M_3+4_ and where vein Cu_1b_ meets wing margin (Fig. 4C, E), cell m_1_ high and narrow (height:width ratio > 2.35). Fore wing longer than 1.6 mm	**10**
–	Fore wing with vein M only slightly arcuate, veins M_1+2_ and M_3+4_ only slightly curved (Fig. 4F), cell m_1_ lower and broader (height:width ratio < 2.2). Fore wing shorter than 1.6 mm. On *Acaciapodalyriifolia*	***Acizziaacaciaebaileyanae***
10	Female proctiger strongly curved downward, with pronounced post-anal bump covered in dense setae, anal ring length ~ 1/3 proctiger length (Fig. 4B). On *Acaciaspirorbis*	***Acizzia* sp. “B**”
–	Female proctiger gradually sloping downward, without post-anal bump, surface with only few, sparse setae, anal ring length ~ 1/2 proctiger length (Fig. 4D). Host plant not confirmed on Norfolk Island, but on *Hakea* and *Grevillea* elsewhere	***Acizziahakeae***
11	Head with genal processes as long or longer than vertex length (Fig. 5A). Fore wing elongate, length ≤ 3 mm, and narrow (ratio fore wing length:width ~ 3.5), apex subacute (Fig. 5D)	***Glycaspisgranulata***
–	Head with genal processes shorter than vertex length, and either broad (Fig. 5B) or otherwise very short, < 1/2 the vertex length (Fig. 5C). Fore wing broader and generally shorter (ratio fore wing length:width 2.4–2.9), apex rounded (Fig. 5E–G)	**12**
12	Larger species; fore wing length always greater than 2 mm (female fore wing often reaching 3 mm), with reddish veins, short pterostigma (~ 0.25 × wing length), long veins M_1+2_ and M_3+4_ making the m_1_ cell value ~ 2 (Fig. 5E). Head with genal processes longer (> 0.5 × vertex length), broader, and contiguous medially (Fig. 5B). On *Eucalyptus* (immature stages producing lerps)	***Cardiaspinafiscella***
–	Smaller species; fore wing length always < 2 mm, with brown veins, longer pterostigma (~ 0.5 × wing length), shorter veins M_1+2_ and M_3+4_ making the m_1_ cell value 1–1.7 (Fig. 5F, G). Head with genal processes shorter (< 0.5 × vertex length) and narrower, diverging or not but not contiguous medially (Fig. 5C). On *Eucalyptus* (immature stages free-living)	**13**
13	Fore wing clear in the center and becoming darker towards the apex, vein Cu_1b_ longer, cu_1_ cell value lower than 2 (Fig. 5F). Head with genal processes diverging (Fig. 5C)	***Blastopsyllaoccidentalis***
–	Fore wing transparent throughout, vein Cu_1b_ shorter, cu_1_ cell value higher than 2 (Fig. 5G). Head with genal processes not diverging	***Cryptoneossatriangula***

**Figure 2.**
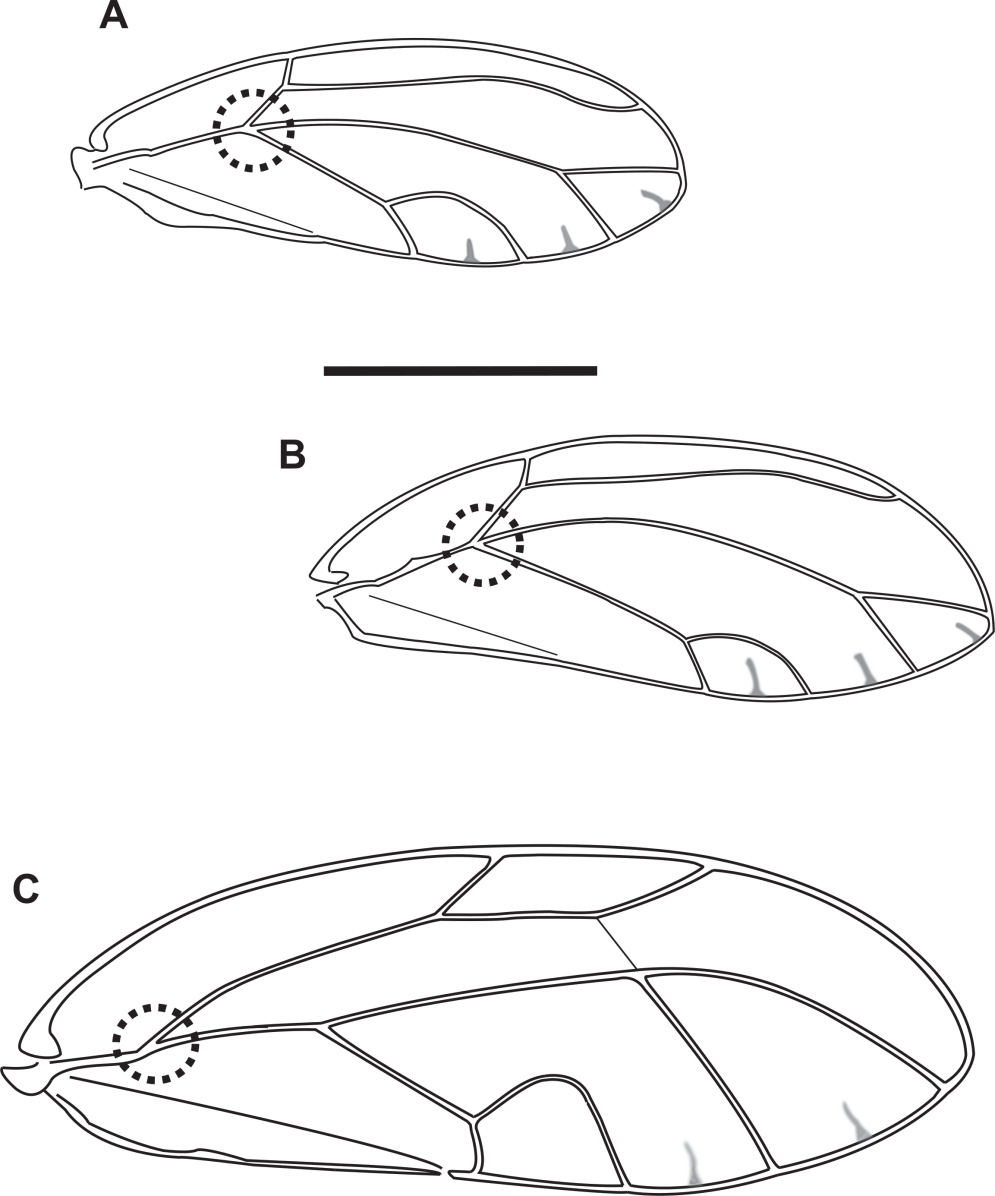
Fore wing (females) **A***Powelliavitreoradiata***B***Bactericeracockerelli***C***Mesohomotomahibisci*. Trifurcation (**A, B**) and bifurcation (**C**) of R+M+Cu stem are circled with a dotted line. Scale bar: 1 mm.

**Figure 3.**
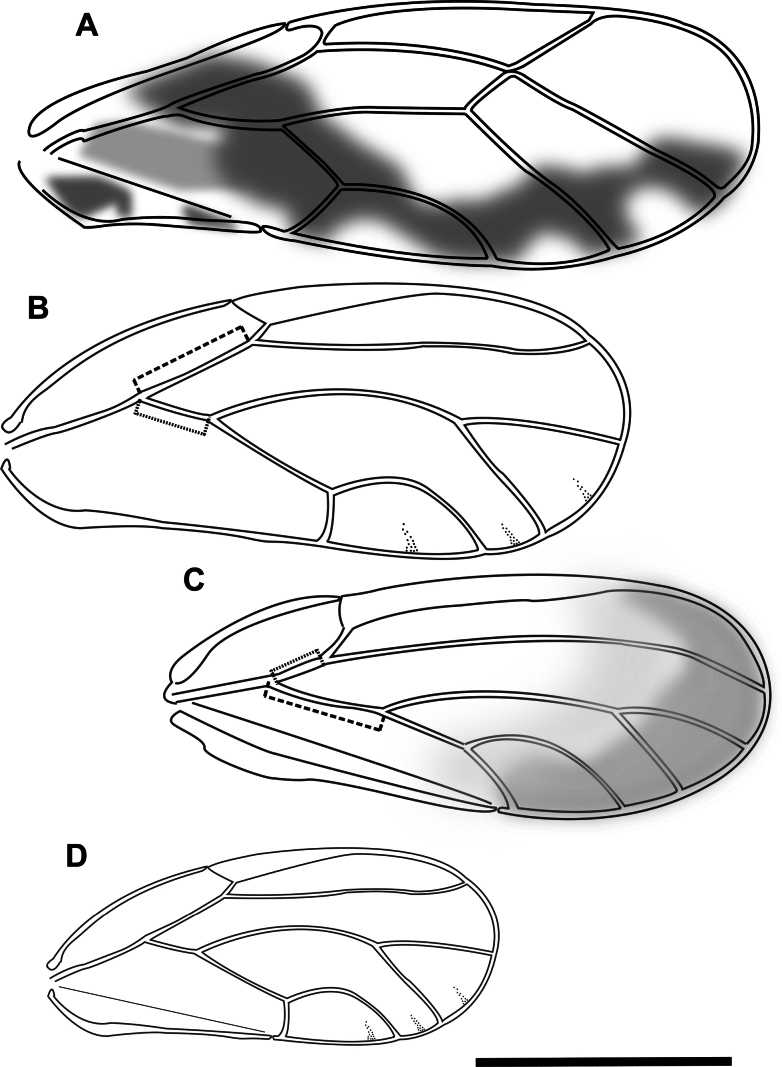
Fore wing (females) **A***Pseudophacopteronaewagriini* Percy & Martoni, sp. nov. **B, D***Heteropsyllacubana* (Psyllidae) **C***Blastopsyllaoccidentalis* (Aphalaridae), showing the different morphology of vein branching, with dashed lines highlighting relative proportion of veins. Scale bar: 1 mm (**A, D** figures **B** and **C** are not to scale)

**Figure 4.**
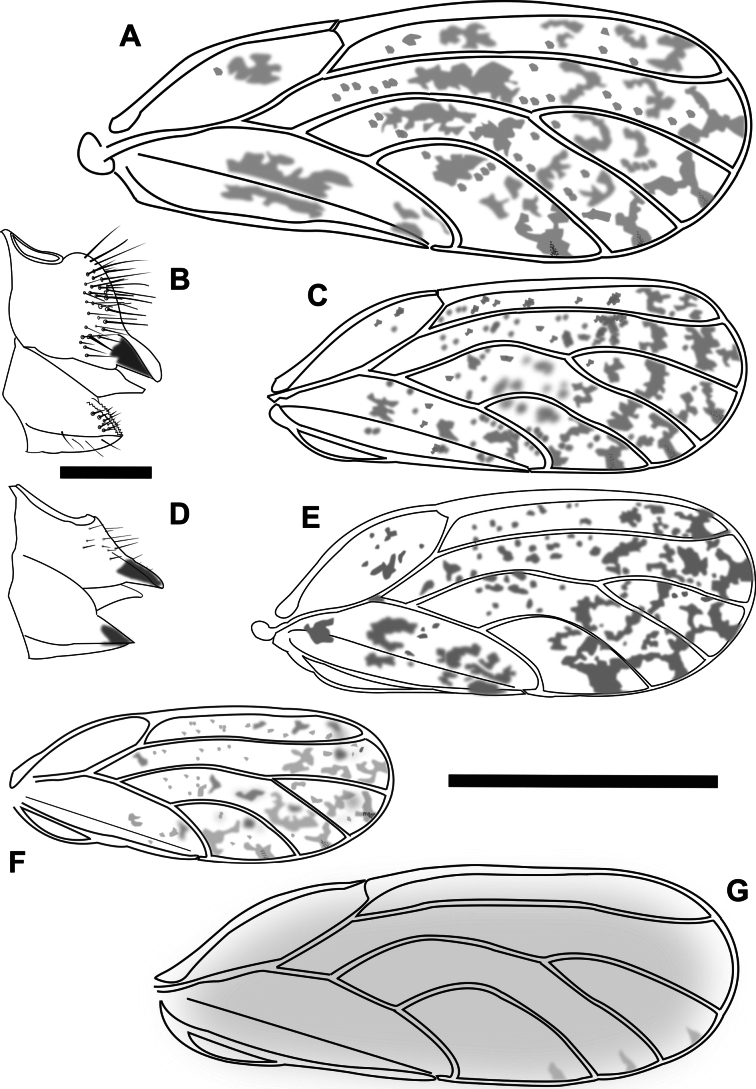
Fore wing and terminalia (females) of Psyllidae species **A***Acizzia* sp. “A” **B**, **C***Acizzia* sp. “B” **D, E***Acizziahakeae***F***Acizziaacaciaebaileyanae***G***Acizziaaliceae* Percy & Martoni, sp. nov. Scale bars: 0.2 mm (**B, D**); 1 mm (**A, C, E–G**).

**Figure 5.**
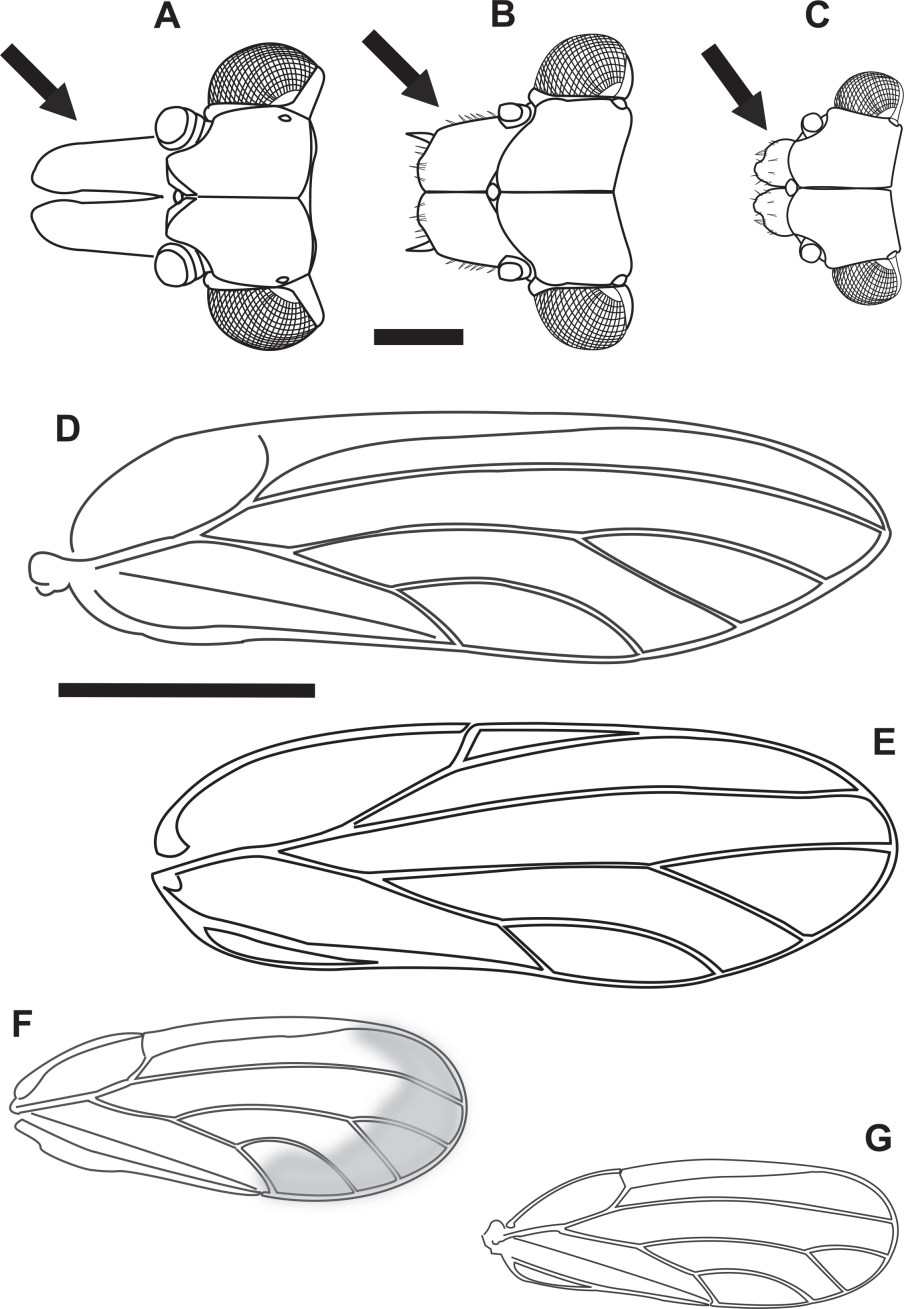
Head (black arrow pointing at length of genal processes, GCL, and of vertex, VL) and fore wings (females) **A, D***Glycaspisgranulata***B, E***Cardiaspinafiscella***C, F***Blastopsyllaoccidentalis***G***Cryptoneossatriangula* (**G**). Scale bars: 0.2 mm (**A–C**); 1 mm (**D–G**).

### ﻿Key to the psyllids of Norfolk Island (5^th^ instar immatures)

**Table:** 

1	Body with a ring of truncate marginal setae present around entire margin of body (Fig. 6A)	**2**
–	Body without a ring of truncate setae on margin of body (Fig. 6F)	**4**
2	Humeral lobes of fore wing pads large, extending almost to anterior margin of eye (Fig. 6B)	**3**
–	Humeral lobes lacking, forewing pads with at most slight anterior bulge (Fig. 8G). Immatures in individually isolated open pit galls on the upper leaf surface of *Alyxiagynopogon* (Apocynaceae) (Fig. 7A–C)	***Pseudophacopteronaewagriini* sp. nov.**
3	Circumanal ring relatively wide and markedly antero-posteriorly constricted (Fig. 6C lower). Immatures, often in aggregates, making shallow pits or depressions on the upper or lower leaf surface of *Pittosporum* spp. (Pittosporaceae) (Fig. 7F)	***Powelliavitreoradiata***
–	Circumanal ring relatively narrow and not markedly antero-posteriorly constricted (Fig. 6C upper). Immatures free-living but can cause leaf distortion and discolouration. On *Solanum* and *Capsicum* spp. (Solanaceae)	***Bactericeracockerelli***
4	Caudal plate distinctly pointed at apex (Fig. 6D). Immatures producing lerps (Fig. 7D). On *Eucalyptus* (*Cardiaspina* and *Glycaspis*)	**5**
–	Caudal plate narrowly or broadly rounded, not distinctly pointed at apex (Fig. 6F–H). Immatures free-living but can be covered in filamentous exudate (Fig. 7E). On *Eucalyptus* or other host plants	**6**
5	Antenna shorter than head width. Shape of lerp bivalve shell-like with woven basket-like construction (Fig. 7D). On *Eucalyptus* (Myrtaceae)	***Cardiaspinafiscella***
–	Antenna longer than head width. Shape of lerp is rectangular, with a dense jumble of filaments that often extend out from the cone. On *Eucalyptus* (Myrtaceae)	***Glycaspisgranulata***
6	Antenna shorter than head width and fore wing pad length (Fig. 6F). Abdominal pores present in small clusters on dorsal surface only. On *Eucalyptus* (*Blastopsylla* and *Cryptoneossa*)	**7**
–	Antenna longer than head width and fore wing pad length (Fig. 12G). Abdominal pores absent or if present, in wide bands on both dorsal and ventral surfaces (Fig. 6H). On other host plants	**8**
7	Smaller species (Fig. 6F left), body length ~ 1 mm, head width ~ 3/4 abdomen width. Antenna length ~ 0.7 × fore wing pad length. Anus with distinct circumanal ring	***Blastopsyllaoccidentalis***
–	Larger species (Fig. 6F right), body length ~ 1.4 mm, head width ~ 2/3 abdomen width. Antenna length ~ 0.5 × fore wing pad length. Anus without distinct circumanal ring (composed of isolated pores)	***Cryptoneossatriangula***
8	Abdomen with 3+3 or 4+4 lanceolate setae on margin (Fig. 6G). On *Leucaena* (Fabaceae)	***Heteropsyllacubana***
–	Abdomen lacking lanceolate setae on margin	**9**
9	Abdomen with anal pores in irregular bands; anus terminal. On *Hibiscustiliaceus* (Malvaceae) (Fig. 6H)	***Mesohomotomahibisci***
–	Abdomen without bands of anal pores; anus ventral (Fig. 12G). On other plants	**10**
10	Abdomen with capitate setae on margin. On *Dodonaea*, *Hakea* or *Grevillea*	**11**
–	Abdomen without capitate setae (at most 1 or 2 pairs of short simple setae) on margin. On *Acacia* (Fabaceae)	***Acizziaacaciaebaileyanae***
11	Abdomen with 4+4 long, slender, narrowly capitate setae on margin (Fig. 12G). Circumanal ring almost horizontal (Fig. 12J). On *Dodonaea* (Sapindaceae)	***Acizziaaliceae* Percy & Martoni, sp. nov.**
–	Abdomen with 6+6 short, robust, broadly capitate setae on margin. Circumanal ring strongly V-shaped. Host plant not confirmed on Norfolk Island, but on *Hakea* and *Grevillea* (Proteaceae) elsewhere	***Acizziahakeae*** ^1^

**Figure 6.**
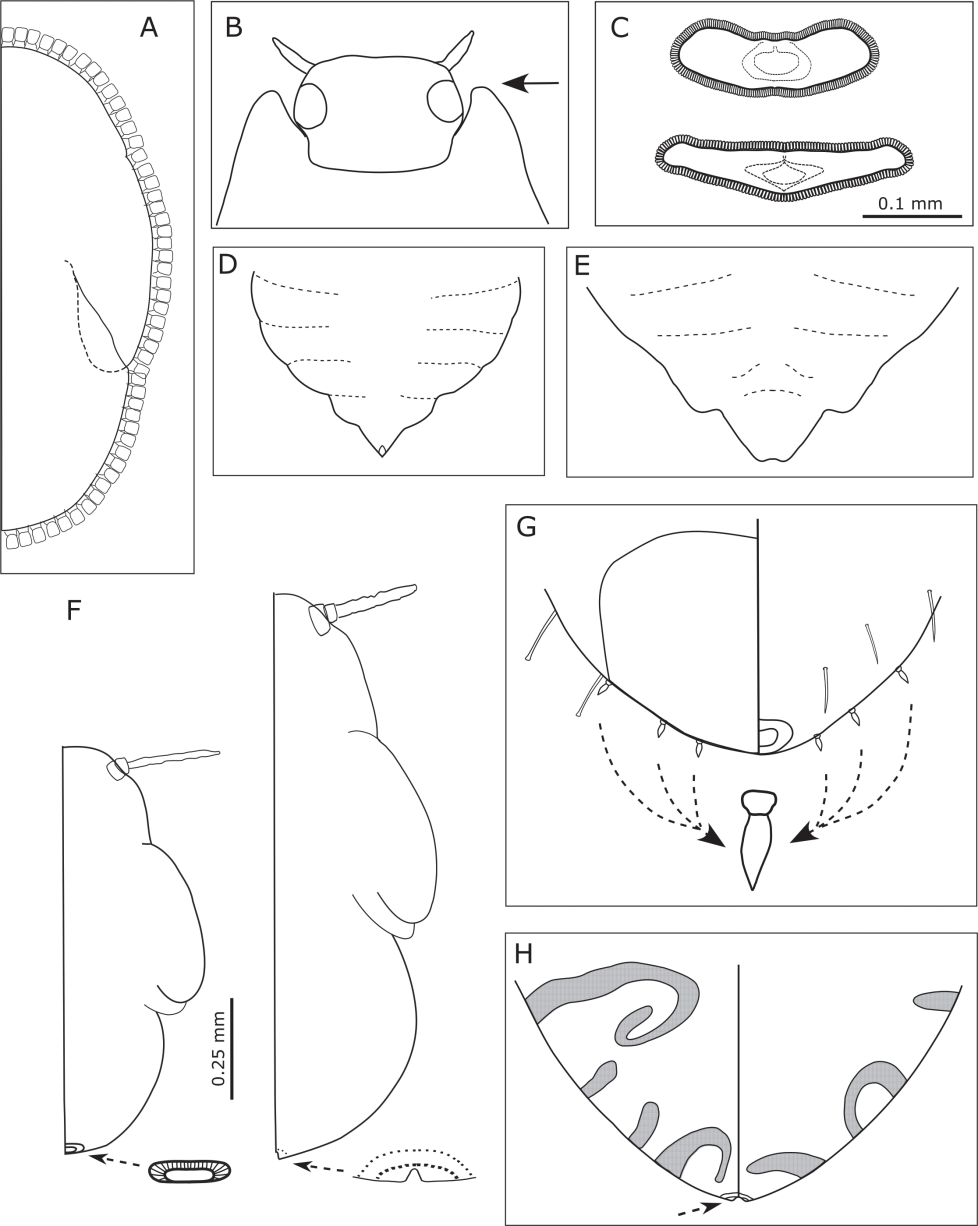
Illustrations of immature characters used in the key (if with longitudinal division, dorsal on left and ventral on right) **A** Example of marginal ring of truncate setae **B** humeral lobes of triozid species **C** circumanal ring shape of *Bactericeracockerelli* (top) and *Powelliavitreoradiata* (bottom) **D** apex of abdomen in *Cardiaspina***E** apex of abdomen in *Glycaspis***F** size and structural difference between *Blastopsylla* (left) and *Cryptoneossa* (right), showing detail of circumanal rings **G** abdomen of *Heteropsyllacubana* showing placement of paired lanceolate setae **H** abdomen of *Mesohomotomahibisci* showing bands of pores and indicating terminal position of anus. Some images redrawn with reference to Taylor (1962, 1985, 1990), White and Hodkinson (1985), and Muddiman et al. (1992). Scale bars: 0.1 mm (**C**); 0.25 mm (**F**).

**Figure 7.**
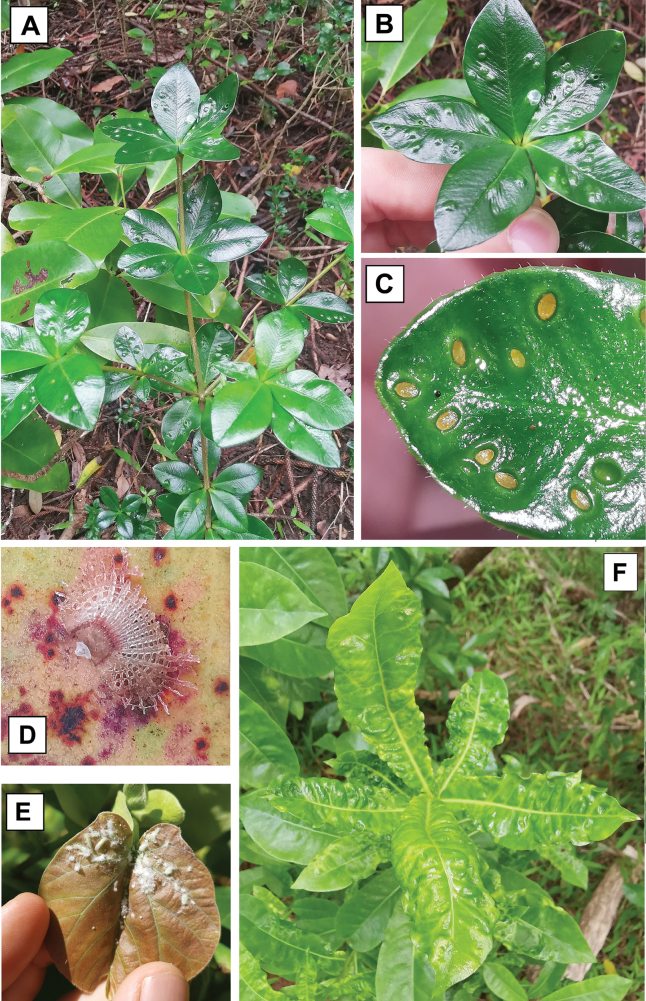
Psyllids on their host plants **A** damage caused by the open pit galls of *Pseudophacopteronaewagriini* Percy & Martoni, sp. nov. on the leaves of *Alyxiagynopogon***B** same, detail of distribution of galls on the upper leaf surface **C** close up of immatures (orange) seated in the pit galls on the upper leaf surface **D** lerp of *Cardiaspinafiscella* on leaves of *Eucalyptus* sp., the lerp is built by the immatures which live underneath until adulthood **E** cluster of both immatures and adults of *Mesohomotomahibisci* on the new growth of *Hibiscustiliaceus* showing production of white waxy filaments **F** damage caused by *Powelliavitreoradiata* on *Pittosporumbracteolatum*, showing the presence of shallow pit galls.

### ﻿Checklist of Norfolk Island Psylloidea

#### ﻿Family Aphalaridae

**Subfamily Phacopteroninae**

##### ﻿ Pseudophacopteron aewagriini

Taxon classificationAnimaliaHemipteraAphalaridae

Percy & Martoni sp. nov.

5A9053FF-3374-5E9F-A9E3-21861972300C

https://zoobank.org/1C9C6FF5-CCB7-4598-A357-EA7B6B05AEC1

Figs 8 , 9 , 10

###### Type locality.

Norfolk Island, Norfolk Island National Park, Red Road Track, on *Alyxiagynopogon* on side of track. Dislodged beating host plant onto tray and collected using entomological aspirator.

###### Type material.

***Holotype*** : Norfolk Island • 1 adult ♂; Norfolk Island National Park, Red Road Track; 17 Oct. 2022; Francesco Martoni leg.; on *Alyxiagynopogon*; sweeping; entire specimen mounted on card triangle, deposited at VAIC. Labels: “Norfolk Island N.P. / Red Road Track / 17-Oct-2022 F. Martoni / On *Alyxiagynopogon*” (printed on white card); “HOLOTYPE ♂ / *Pseudophacopteronaewagriini* / Percy and Martoni 2025” (printed on red card). ***Paratypes***: Norfolk Island • 5 adult ♂♂, 5 adult ♀♀; same data as the holotype, dissected specimens mounted on microscope slides, deposited at VAIC • 1 adult ♂, 2 adult ♀♀; same data as the holotype, entire specimens mounted on card triangle, deposited at ANIC • 16 adult ♂♂, 18 adult ♀♀; Norfolk Island National Park, Bridle Track; 15 Oct. 2023; James M.H. Tweed leg.; on *Alyxiagynopogon*; entire specimens preserved in ethanol, deposited at VAIC • 2 adult ♂♂, 2 adult ♀♀, same as for preceding; entire specimens preserved in ethanol, deposited at NHMB • ~ 200 immatures; Norfolk Island National Park, Red Road Track car park; 08 Nov. 2023; James M.H. Tweed leg.; on *Alyxiagynopogon*; entire specimens preserved in ethanol, deposited at VAIC • 1 adult ♂, 3 adult ♀♀; Norfolk Island National Park, Forbidden Track; 20 Feb. 2023; James M.H. Tweed leg.; On *Alyxiagynopogon*; entire specimens preserved in ethanol, deposited at VAIC • 3 adult ♂♂; Norfolk Island National Park, Red Road Track; 15 Oct. 2022; Francesco Martoni leg.; on *Alyxiagynopogon*; entire specimens preserved in ethanol, deposited at VAIC • 4 adult ♂♂; Norfolk Island National Park, Palm Glen; 11 Jul. 2013; Alice Wells leg.; on *Alyxiagynopogon*; AW-12-95; entire specimens preserved in ethanol, deposited at DMPC • 3 adult ♂♂, 2 adult ♀♀,; Norfolk Island National Park, Palm Glen; 22 Oct. 2013; Laurence Mound leg.; on *Alyxia* sp.; LAM5815; entire specimens preserved in ethanol, deposited at DMPC. All paratypes are labelled as “PARATYPE ♂-♀ / *Pseudophacopteronaewagriini* / Percy & Martoni 2025” (printed on blue card).

###### Other material examined.

Norfolk Island • ~ 20 immatures; Norfolk Island National Park, Red Road Track; 14 Mar. 2022; Francesco Martoni leg.; on *Alyxiagynopogon*; entire specimens preserved in ethanol, deposited at VAIC. Not included in the type series because they were damaged during a semi-destructive DNA extraction protocol.

###### Diagnosis.

The shape of the fore wing of *P.aewagriini*, which is elongate and narrow (> 2.6 × longer than wide), as well as the pigmentation pattern, clearly aligns this taxon with other *Pseudophacopteron* in the Austro-Pacific region (Malenovský 2008); the type species, *P.tuberculatum* Crawford, as well as most other taxa also found on Apocynaceae host plants, have a fore wing that is pyriform and broad (length < 2.6 × width) (Malenovský 2008; Malenovský and Burckhardt 2009; Malenovský et al. 2015). A similar narrow wing morphology to that of *P.aewagriini* can be observed in some of the African *Pseudophacopteron* species such as *P.nigritulum* Malenovský and Burckhardt and *P.wagneri* Malenovský and Burckhardt (Malenovský and Burckhardt 2009), but in these cases the vertex lacks a distinct median ridge, and only *P.wagneri* is possibly associated with Apocynaceae. See also the Remarks section below.

###### Description.

**Figure 8.**
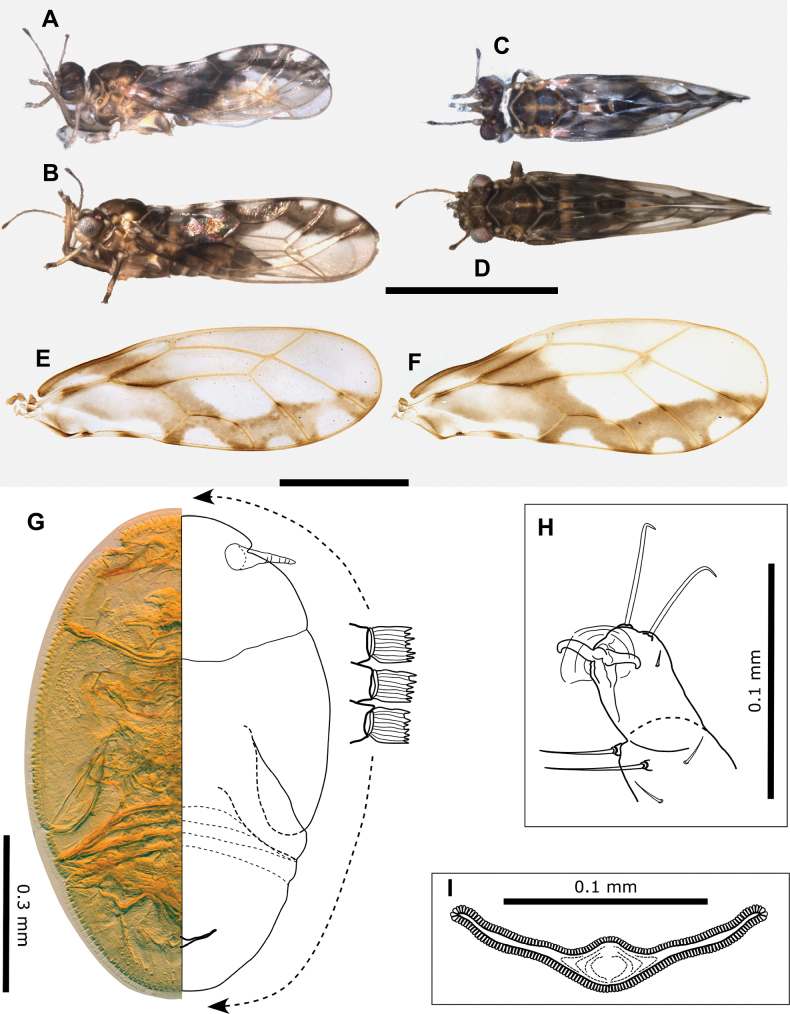
*Pseudophacopteronaewagriini* Percy & Martoni, sp. nov. **A** adult lateral habitus of male **B** same, female **C** dorsal habitus of male **D** same, female **E** fore wing of male **F** same, female **G** immature habitus showing type of truncate marginal setae **H** metatibiotarsus of immature **I** circumanal ring of immature. Scale bars: 1 mm (**A–D**); 0.5 mm (**E, F**); 0.3 mm (**G**); 0.1 mm (**H, I**).

**Figure 9.**
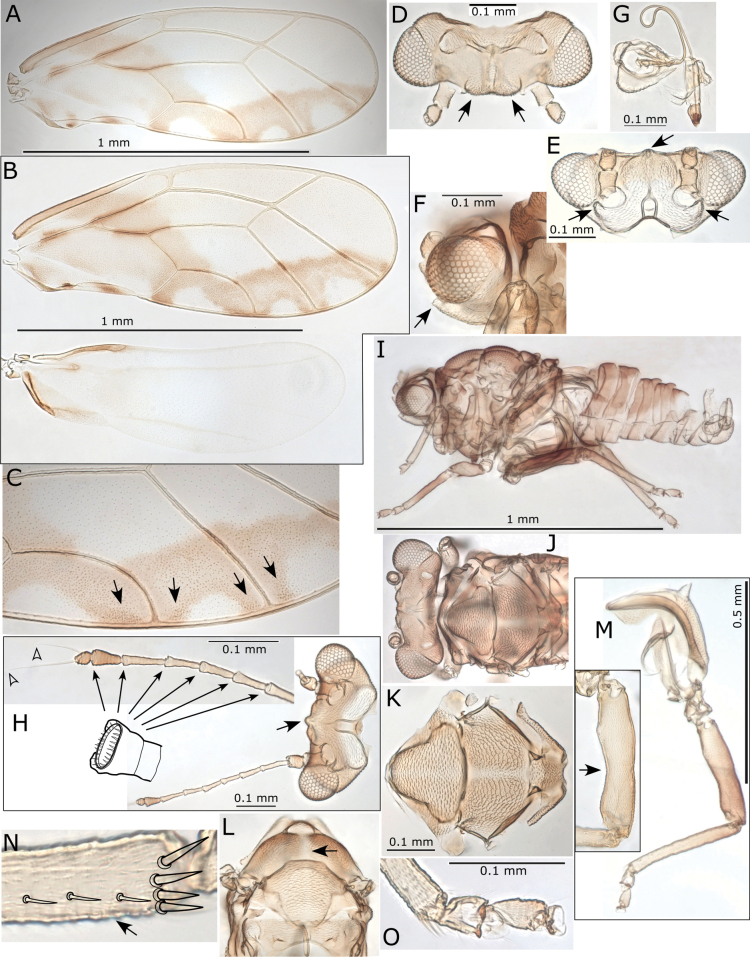
*Pseudophacopteronaewagriini* Percy & Martoni, sp. nov. **A** fore wing of male **B** fore wing and hind wing of female **C** detail of radular spinule cluster positions **D** head (dorsal view) indicating anterior bulges either side of median ridge **E** head (anterior view) indicating median ridge and genal tubercles below toruli **F** head (lateral view) indicating protruding genal tubercles **G** clypeus and proboscis **H** head and antennae (with detail inset) indicating median ridge on vertex and position of large rhinaria on segments 4–9, termination of two apical setae is indicated by open arrow heads **I** male (lateral view) **J** head and thorax (dorsal view) **K** thorax from dorsal view **L** thorax from anterior view indicating medial depression on mesoscutum **M** hind leg with detail of medial constriction of metafemur (inset) **N** metatibia apex indicating relatively slender lateral setae **O** metatarsi showing two metabasitarsus spines.

***Colouration*.** Adult. Head pale brown. Antennae with segments 1 and 2 brown, segments 3–8 of a very pale brown, and segments 9 and 10 dark brown tending to black. Thorax mostly dark brown but with medial line crossing mesoscutum and mesopraescutum lighter. Legs with dark brown femora and basal part of tibiae, and with pale brown apical part of tibiae and tarsi (Fig. 8A–D). Fore wings hyaline, with dark brown pattern covering basal 1/3 of wing and reaching wing apex in the basal portion of cell r_2_ as a band along posterior wing margin, leaving small transparent areas in cells cu_2_, cu_1_, m_1_ and m_2_; dark pattern also covering the proximal part of fore wing, reaching the vein C+Sc, crossing the middle of cell c+sc and the bifurcation of stem R+M+Cu (Figs 8E, F, 9A, B); fore wing veins pale brown or dark brown in areas covered by dark pattern. Hind wing pale to darker brown basally (Fig. 9B). Male and female terminalia pale brown to pale yellow. Female proctiger darker on the tip.

Immature (5^th^ instar). Body uniformly dark yellow-brown (Fig. 8G).

***Structure*.** Adult. Body relatively small, 1.5–1.9 mm from anterior margin of vertex to tip of folded wings (males smaller than females) (Fig. 8A–D). Head (Figs 8A–D, 9D–F, H–J) in lateral view not deflexed downward and held in same plane as body, wider than thorax but narrower than antennal length, vertex width almost 3 × length, with prominent narrow median ridge and two bulges anteriorly either side of ridge, lateral ocelli raised on small tubercles above the plane of vertex, median epicranial suture reduced. Genae (Fig. 9E, F) small, weakly swollen, genal tubercles below toruli small and acute, prominent in lateral profile. Clypeus (Fig. 9G) subglobular, terminal proboscis segment short. Antenna (Fig. 9H) with ten segments, segment 3 longest with segments 4–8 of subequal length, widening slightly from base to apex, segment 9 slightly shorter and wider apically, while segment 10 very short, < 1/2 the length of segments 3–8 and ~ 1/2 the length of segment 9; a single subapical rhinarium on each of segments 4–9, large, elliptical and fringed by cuticular spines; terminal setae unequal, with longer seta reaching 0.1 mm, shorter seta between 1/2–2/3 length of longer and approx. as long as segments 9 and 10 combined.

Thorax (Fig. 9I–K) moderately arched, mesoscutum with reduced microsculpture and pigmentation in medial depression. Mesotibia with subapical comb of ≤ 6stout setae. Hind legs (Fig. 9M–O) with small, acute and slightly curved meracanthus; metafemur constricted medially, length subequal to metatibia; metatibia without genual spine basally, with an open crown of seven unsclerotised spurs apically and ≤ 12 stout lateral setae more slender than apical spurs; metabasitarsus subglobular, approx. as long as broad, slightly shorter than apical tarsus, with two sclerotised lateral spurs.

Fore wing (Figs 8E, F, 9A, B) elongate, 2.6–2.8 × longer than wide, much wider in apical half, rounded at apex; cell m_1_ narrower and more elongate than cell cu_1_, vein M approx. as long as vein Rs to the point it meets vein M_1+2_ and only slightly arched; membrane with dispersed spinules distributed in all cells and with spinule-free bands along veins, radular spinules concentrated into small triangular fields in outer pigmented corners of apical cells cu_1_, m_2_, m_1_, and the adjacent corner of r_2_ (Fig. 9C).

**Figure 10.**
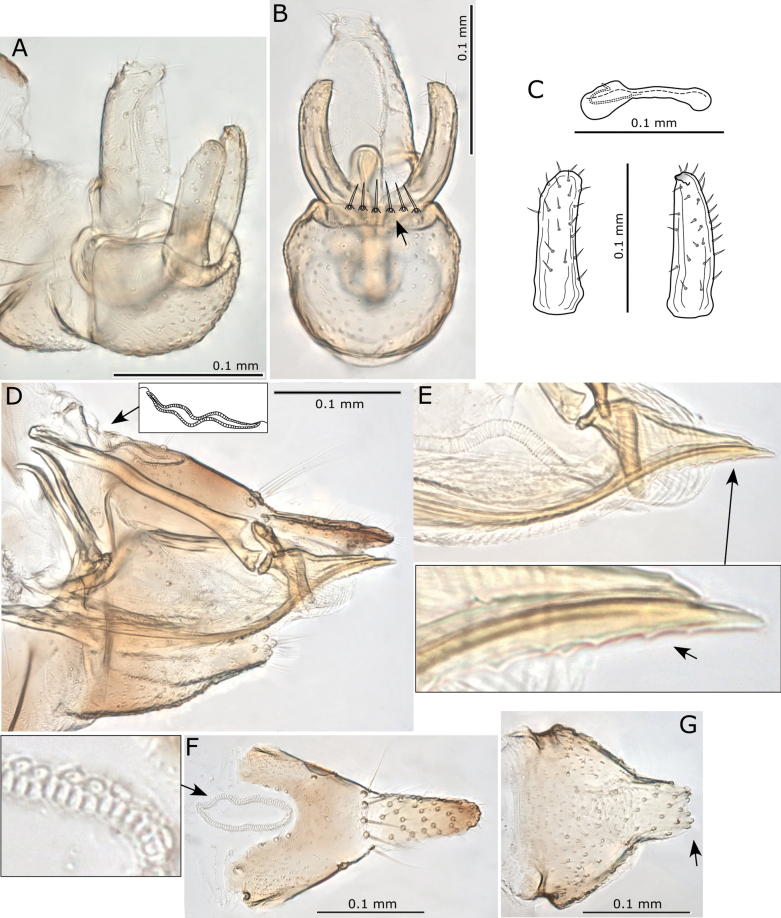
*Pseudophacopteronaewagriini* Percy & Martoni, sp. nov. **A** male terminalia (lateral view) **B** male terminalia (posterior view) indicating a row of stout setae on dorsal margin **C** paramere outer surface (left) and inner surface (right), and aedeagus **D** female terminalia (lateral view) showing irregular profile of anal ring (inset) **E** ovipositor (lateral view) showing detail of shallow serrations on valvulae dorsalis **F** female proctiger (dorsal view) with detail of anal ring pores **G** female subgenital plate (ventral view) indicating truncate and slightly incised apex.

Male terminalia (Fig. 10A–C) with subgenital plate subglobular, dorsal margin slightly sinuate and posteriorly bearing several long stout setae; proctiger relatively slender, cylindrical, in lateral profile straight except apex which is slightly bent posteriorly; parameres simple, shorter than proctiger, in lateral profile parallel sided and more or less straight, apex bluntly rounded and slightly bent posteriorly, inner surface with a weakly produced and marginally sclerotized tooth subapically; outer and inner surface covered with fine setae and a few slightly stouter setae subapically. Distal segment of aedeagus relatively short, apical portion ~ 1/3 as long as the distal segment length, somewhat elongate, broadly globular, unhooked but angled downward, apex rounded.

Female terminalia (Fig. 10D–G) with proctiger much longer than subgenital plate, narrowing to bluntly acute apex covered in stout setae, anal ring narrowly oval with the outline in profile and dorsal view irregular, composed of a double row of intermittently irregular pores, anal ring length ~ 1/3 proctiger length; subgential plate ~ 2/3 proctiger length, narrowing to a truncate, weakly incised apex; ovipositor dorsal valvulae triangular, ventral valvulae finely serrate with a series of shallow teeth ventrally.

Immature (5^th^ instar): body ~ 1.75 × as long as wide, relatively large in size compared to the adult, shape narrowly oval, dorso-ventrally flattened, wing pads not protruding (Fig. 8G). Entire margin of head, wing pads and abdomen ringed with longitudinally ridged, truncate marginal setae with apices raggedly uneven (Fig. 8G). Antenna short, length ~ 0.32–0.45 × head width, with ~ 7 indistinct subdivisions (rhinaria not clearly visible). Fore wing pads lacking distinct humeral lobes, but with slight anterior bulges. Tarsal arolium shorter than claws, membranous, fan-shaped with unguitractor, claws well developed (Fig. 8H). Anus in ventral position (Fig. 8G), circumanal ring shallowly V-shaped, antero-posteriorly constricted and slightly sinuous, consisting of a single row of elongate pores (Fig. 8I).

***Measurements*** (in mm). Adults (5 ♂♂, 5 ♀♀). Length of body (vertex to terminalia) ♂ 1.01–1.17, ♀ 1.22–1.35; length of body (vertex to apex of folded wings) ♂ 1.55–1.80, ♀ 1.78–1.93; width of head (HW) ♂ 0.37–0.42, ♀ 0.40–0.45; length of antenna (AL) ♂ 0.40–0.44, ♀ 0.47–0.51; longer antennal terminal seta length (T1) ♂ 0.06–0.09, ♀ 0.07–0.10; shorter antennal terminal seta length (T2) ♂ 0.03–0.05, ♀ 0.04–0.06; length of antennal segments 9 and 10 together (TS) ♂ 0.05–0.06, ♀ 0.06–0.07; length of fore wing (WL) ♂ 1.27–1.40, ♀ 1.40–1.57; width of fore wing (WW) ♂ 0.45–0.52, ♀ 0.51–0.58; length of line connecting base of vein C+Sc and apex of vein R_1_ (CS) ♂ 0.51–0.58, ♀ 0.59–0.65; length of line connecting base of vein C+Sc and costal break (CB) ♂ 0.46–0.52, ♀ 0.51–0.59; length of line connecting the touching point of veins Rs and M_1+2_ and apex of Rs (a) ♂ 0.17–0.21, ♀ 0.20–0.25; length of line connecting the touching point of veins Rs and M_1+2_ and apex of M_1+2_ (b) ♂ 0.41–0.45, ♀ 0.47–0.54; length of line connecting apices of veins Rs and M_1+2_ (c) ♂ 0.36–0.41, ♀ 0.40–0.48; length of line connecting apices of veins Cu_1a_ and Cu_1b_ (d) ♂ 0.34–0.38, ♀ 0.38–0.44; length of line connecting base and apex of vein Cu_1b_ (e) ♂ 0.12–0.14, ♀ 0.13–0.16; metatibia length (TL) ♂ 0.25–0.31, ♀ 0.28–0.35; male proctiger length (MP) ♂ 0.10; paramere length (PL) ♂ 0.08–0.10; length of distal segment of aedeagus (DL) ♂ 0.08–0.09; female proctiger length (FP) ♀ 0.23–0.27; female subgenital plate length (SL) ♀ 0.14–0.19. Ratios: AL:HW ♂ 1.00–1.19, ♀ 1.13–1.19; T1:TS ♂ 1.20–1.60, ♀ 1.14–1.17; T1:T2 ♂ 1.60–2.00, ♀ 1.50–1.75; WL:HW ♂ 3.28–3.43, ♀ 3.49–3.58; WL:WW ♂ 2.69–2.84, ♀ 2.64–2.80; CB:CS ♂ 0.86–0.91, ♀ 0.86–0.91; a:b ♂ 0.40–0.51, ♀ 0.39–0.52; a:c ♂ 0.45–0.58, ♀ 0.44–0.63; d:e ♂ 2.64–2.83, ♀ 2.56–3.00; TL:HW ♂ 0.68–0.74, ♀ 0.70–0.78; MP:HW ♂ 0.24–0.27; PL:HW ♂ 0.19–0.24; DL:HW ♂ 0.21–0.22; FP:HW ♀ 0.57–0.63; SL:FP ♀ 0.35–0.42.

Immatures (5^th^ instar, *n* = 4). Length of body 1.50–1.60; width of body 0.88–0.96; length of antennae 0.17–0.25; width of head 0.54–0.56.

###### Etymology.

The name epithet uses the Norf’k (local resident language spoken on Norfolk Island) word “aewagriin” that refers to the host plant, *Alyxiagynopogon*, known on Norfolk Island as the Evergreen. The name is treated as a Latinised noun, gender masculine, in genitive case. This name was chosen by receiving multiple nominations during the Norfolk Island Flora and Fauna Society meeting, held on Norfolk Island on the 10 June 2023. Members of the society remarked on the importance of such a species that has managed to “hairng orn” (hold its place) on Norfolk Island.

###### Distribution.

This species is the only *Pseudophacopteron* present on Norfolk Island, and has been recorded from locations throughout Norfolk Island National Park (Fig. 15). The species has also been recorded within Selwyn Reserve, a Norfolk Island Regional Council reserve which adjoins the western border of the National Park (G. Maynard, pers. comm. 2024; Fig. 15). The distribution of this species is limited by the distribution of the host plant, which although common within the National Park, is scarce across the rest of the island group. No specimens have been found on the few plants that have been located and inspected outside of the National Park and Selwyn Reserve and it is likely *P.aewagriini* is confined to the National Park and its immediate surrounds.

###### Host plant on Norfolk Island.

*Alyxiagynopogon* Roem. & Schuit. (Gentianales, Apocynaceae).

###### Conservation.

This species is considered endemic to Norfolk Island, as is its host plant, *Alyxiagynopogon*. The host is not currently regarded as threatened, however, it is almost entirely confined to Norfolk Island National Park, with only scattered specimens known from other areas of the island. Neither species is known from nearby Phillip Island. Despite searching, *P.aewagriini* is known only from the National Park and the adjoining Selwyn Reserve, meaning the EOO ranges from 1.3–6.8 km^2^, based on either confirmed occurrence records, or the entire area of the National Park, Botanic Gardens, and Selwyn Reserve (Fig. 15). The corresponding AOO calculated using a 4 km^2^ grid overlay, ranges from 16–20 km^2^. The range reduction suffered by this species following European colonisation is likely to have ceased in 1984 following the establishment of the National Park. It is likely that *A.gynopogon* and *P.aewagriini* increased in range slightly following the cessation of grazing by cattle within the National Park. The species is not known to be undergoing a population or range decline, nor is it known to be facing any ongoing threats. However, Norfolk Island National Park and Selwyn Reserve have been identified as being highly vulnerable to wildfire under optimal fire conditions which could destroy a large proportion of the habitat for *P.aewagriini*, particularly given Norfolk Island’s ecosystems are not fire-adapted (Eco Logical Australia 2021). Predicted drying of Norfolk Island’s climate (Petheram et al. 2020) exacerbates the risk of wildfire, and may also reduce the quality of the rainforest inhabited by this species and its host plant. Invasive Argentine ants (*Linepithemahumile*) are established on the island and although they are subject to an extensive control and eradication effort (NIRC 2021) their spread into the National Park could impact *P.aewagriini* populations.

We propose that *P.aewagriini* warrants a threat classification of Vulnerable under criterion D2 (IUCN 2012). This species is known from only a single location but is not currently known or suspected to be undergoing continuing decline or extreme fluctuation in its range or population size. However, it has an EOO of 16–20 km^2^ and plausible future threats in the form of wildfire which could destroy much of its habitat, ongoing reduction of habitat quality due to predicted climate drying, and Argentine ant invasion. As such, this species could quickly be driven to Critically Endangered under Criteria B if these threats were to materialise (IUCN 2012). Consideration should be given to establishing populations of *A.gynopogon* and *P.aewagriini* in other areas of Norfolk Island, as well as on nearby Phillip Island, to increase the security of both species.

###### DNA resources.

GenBank COI: MG988815, MG988814, cytB: MG989134, MG989135. Also represented in the mitogenome analysis of Percy et al. (2018) as: DP1.idba.137, and the annotated mitochondrial genome is in GenBank: MG989234 (Percy et al. 2018; see also Fig. 16). Additionally, a total of four COI sequences were generated for this study (OR558312–OR558315; Table 1).

###### Systematics.

This species may be related to *Pseudophacopterontuberculatum* (Crawford, 1912) which is native to China, southeast Asia, and Papua New Guinea (PNG) and induces closed galls on the leaves of *Alstonia* (Apocynaceae; Percy et al. 2016; Luo et al. 2018), the same plant family as *Alyxia*. Although Luo et al. (2018) noted that there are no morphological characters suggesting that all *Pseudophacopteron* species developing on Apocynaceae constitute a monophyletic group, the molecular data do support *P.aewagriini* from Norfolk Island and *P.tuberculatum* (COI GenBank sample MH769661) as likely being in the same clade. Unfortunately, the backbone analysis currently involves inclusion of more short (and non-overlapping) sequences than there are taxa in the original mitogenome data, which makes a rigorous backbone analysis not possible at this time. Morphologically, there are some shared traits with two species described from Brazil, *P.aspidospermi*Malenovský et al., 2015 and *P.longicaudatum*Malenovský et al., 2015, which produce closed leaf galls on *Aspidosperma* (Apocynaceae) (Malenovský et al. 2015); but overall, more systematic data on this group in the Austro-Pacific region are needed for conclusions to be made.

###### Remarks.

This species is the first described *Pseudophacopteron* species known to be associated with *Alyxia* (Apocynaceae). The related *Pseudophacopterontuberculatum* is considered a serious pest of plantations of *Alstoniascholaris* (Apocynaceae) in the Philippines (Braza and Calilung 1981). The host plant of *P.aewagriini*, *Alyxiagynopogon*, is a Norfolk Island endemic, evergreen understory shrub, which is relatively common within the National Park. The galling of leaves by *P.aewagriini*, inducing the characteristic open pit galls on the leaves (Fig. 7A–C), does not appear to be overly detrimental to the host. Hollis (2004) and Malenovský (2008) reported the presence of an unnamed species of *Pseudophacopteron* on Lord Howe Island, where there are three species of *Alyxia*: the endemic *A.lindii* and *A.squamulosa*, and the native *A.ruscifolia*. A number of *Pseudophacopteron* species associated with *Alyxia*, not yet formally described, have been reported from Western Australia (iNaturalist observation 138485676), Queensland (Malenovský 2008, iNaturalist observation 154392985) and South Australia (G.S. Taylor, pers. comm. 2023). Informal descriptions of two of these species were included in the PhD thesis of Malenovský (2008). These two species are close to *Pseudophacopteronaewagriini* and all three species belong to the same “narrow-winged” clade identified by Malenovský (2008), which also includes a *Pseudophacopteron* sp. from Papua New Guinea, another species from both Papua New Guinea and Sulawesi, and a third species from Costa Rica (Malenovský 2008). Malenovský (2008) also noted that the two species from Queensland and Lord Howe Island can be separated from the remaining taxa in the “narrow-winged” clade by the presence of a distinct median ridge and anterior bulges or tubercles on the vertex, and these characteristics are shared with *P.aewagriini* (Fig. 9D, E, H). *Pseudophacopteronaewagriini* can be differentiated from the undescribed taxa occurring in Queensland and Lord Howe Island in the relative length of the paired apical antennal setae, with the two undescribed species showing these setae to be of similar or slightly unequal length, while in *P.aewagriini* the shorter seta is between 1/2–2/3 length of the longest seta (Fig. 9H). The fore wing of *P.aewagriini* is distinct in appearing intermediate between the two undescribed species. The overall fore wing structure and shape of *P.aewagriini* is more similar to the Queensland species but is less narrow as in the Lord Howe Island species. The basal fore wing markings are more similar to that of the Lord Howe Island taxon, without a distinct unpigmented area alongside vein M+Cu, but more similar to the Queensland taxon in the marginal fore wing markings which have larger unpigmented spots in cells cu_1_, m_1_ and m_2_. The extent of marginal pigmentation in cell r_2_ is reduced in *P.aewagriini* compared to either of the undescribed Australian species. Additionally, *P.aewagriini* shares the seven unsclerotised apical spurs on the metatibia with the Lord Howe Island taxon, versus 11 in the Queensland taxon.

#### ﻿Subfamily Spondyliaspidinae

##### *Blastopsyllaoccidentalis* Taylor, 1985

Fig. 11A

**Distribution.** Native to Australia (Taylor 1985) and previously reported on Norfolk Island (Maynard et al. 2018). Present also in the Americas – Argentina (Bouvet et al. 2005), Brazil (Burckhardt et al. 1999), Chile (Burckhardt and Elgueta 2000), Mexico (Hodkinson 1991), Nicaragua (Queiroz et al. 2018), United States of America (California – Taylor 1985; Florida – Halbert et al. 2001), Uruguay (Martínez et al. 2014); Africa – Burundi (Queiroz et al. 2018), Cameroon (Dzokou et al. 2009), Egypt (El Nasr and Abd-Rabou 2012), Kenya (Hollis 2004), South Africa (Neser and Millar 2007); Asia – China (Hollis 2004), Indonesia (Borneo – Burckhardt et al. 2024; Sumatra – Queiroz et al. 2018), Israel (Spodek et al. 2015), Philippines, Turkey (Drohojowska and Burckhardt 2014), Yemen (Queiroz et al. 2018); Europe – Cyprus (Demetriou et al. 2022), Italy (Laudonia 2006), Malta (Mifsud and Carapezza 2020), Portugal, Spain (Pérez-Otero et al. 2011); Oceania – New Zealand (Dale 1985).

**Host plant on Norfolk Island.***Eucalyptusbotryoides* Sm. (Myrtales, Myrtaceae), confirmed by the collection of immatures (Suppl. material 1).

**Remarks.** Adventive to Norfolk Island (Maynard et al. 2018). Immature stages are free-living.

##### *Cardiaspinafiscella* Taylor, 1962

Fig. 11B

**Figure 11.**
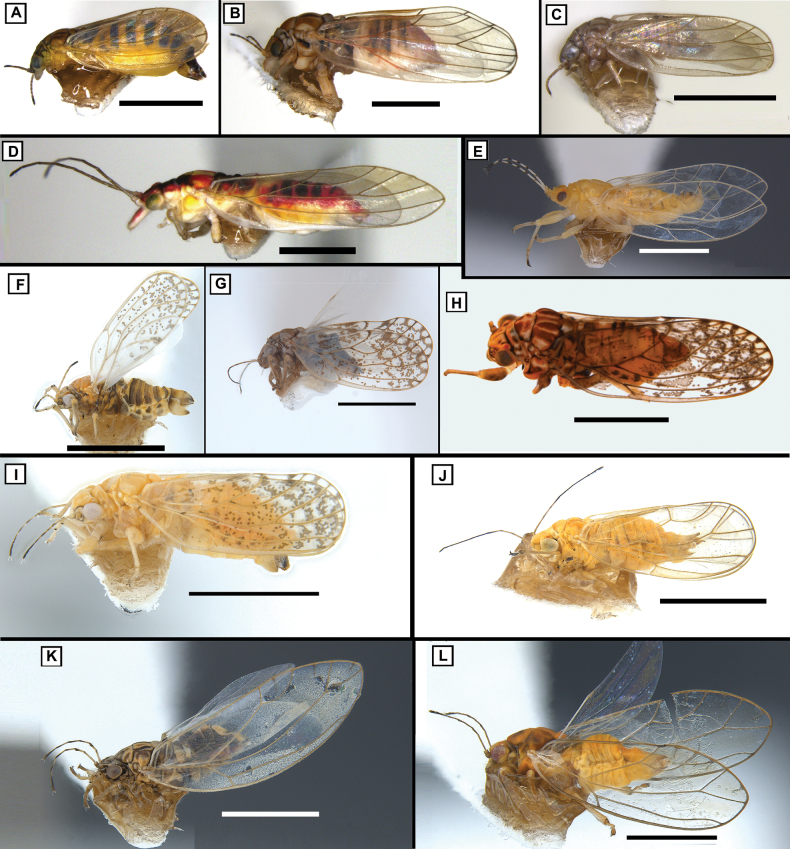
Lateral habitus (all female except where noted) **A***Blastopsyllaoccidentalis***B***Cardiaspinafiscella***C***Cryptoneossatriangula***D***Glycaspisgranulata***E***Mesohomotomahibisci* (male) **F***Acizziaacaciaebaileyanae***G***Acizziahakeae***H***Acizzia* sp. B **I***Acizzia* sp. A **J***Heteropsyllacubana***K***Bactericeracockerelli***L***Powelliavitreoradiata*. Scale bars: 1 mm.

**Distribution.** Native to Australia (Taylor 1962) and previously reported on Norfolk Island (Maynard et al. 2018). Present also in New Zealand (Henderson et al. 2010)

**Host plant on Norfolk Island.***Eucalyptusbotryoides* (Myrtales, Myrtaceae). No immature specimen was collected (Suppl. material 1), but lerps were observed (Fig. 7D).

**Remarks.** Adventive to Norfolk Island (Maynard et al. 2018). The immatures of this species are known to build lerps (Taylor 1962), and these were recorded on Norfolk Island (Fig. 7D).

##### *Cryptoneossatriangula* Taylor, 1990

Fig. 11C

**Distribution.** Native to Australia (Taylor 1990). Present in New Zealand (Henderson et al. 2010) and United States of America (California – Gill 1995, Brennan et al. 1999; Oregon – Castillo Carrillo et al. 2016).

**Host plant on Norfolk Island.***Eucalyptus* sp. (Myrtales, Myrtaceae).

**Remarks.** First report from Norfolk Island, where it is considered adventive. The immatures of this species are free-living, but no immature specimen was collected on Norfolk Island.

##### Glycaspis (Glycaspis) granulata (Froggatt, 1901)

Fig. 11D

*Spondyliaspisgranulata* Froggatt, 1901: 293.

*Glycaspisgranulata*; Taylor 1960: 385.

Glycaspis (Alloglycaspis) granulata; Moore 1961: 164–165.

Glycaspis (Glycaspis) granulata; Moore 1970: 308.

**Distribution.** Native to Australia (Froggatt 1901) and previously reported on Norfolk Island (Maynard et al. 2018). Present in New Zealand (Withers 2001).

**Host plant on Norfolk Island.***Eucalyptus* sp. (Myrtales, Myrtaceae).

**Remarks.** Adventive to Norfolk Island (Maynard et al. 2018). The immatures of this species are known to build square-shaped white lerps (Moore 1970), although no lerps were observed on the island during the collection of the specimens.

#### ﻿Family Carsidaridae

**Subfamily Carsidarinae**

##### *Mesohomotomahibisci* (Froggatt, 1901)

Fig. 11E

*Tyorahibisci* Froggatt, 1901: 287.

*Udamostigmahibisci*; Enderlein 1910: 138.

*Mesohomotomahibisci*; Crawford 1920: 356.

**Distribution.** Originally described from Australia (Froggatt 1901) and previously reported on Norfolk Island (Maynard et al. 2018). Present in Oceania – Bismarck Archipelago, Caroline Islands, Cook Islands, Fiji (Hodkinson 1983), French Polynesia (including Marquesas – Hollis 1987, Society and Austral Islands – Hodkinson 1983), Gilbert Islands, New Caledonia, Palau, Solomon Islands, Tonga, Vanuatu (Hodkinson 1983); Africa – Cameroon (Yana et al. 2015), Democratic Republic of the Congo, Kenya, Madagascar, Mauritius, Seychelles, South Africa, Tanzania, Uganda, Zimbabwe (Burckhardt and van Harten 2006); Asia – Chagos Archipelago (Hodkinson 1983), China (Hong Kong – Hodkinson 1986), India, Indonesia (Burckhardt and van Harten 2006; including Borneo – Hodkinson 1983), Japan (Kyushu – Hodkinson 1983; Ryukyu Islands – Burckhardt and van Harten 2006), Laos (Cho et al. 2017), Malayasia (peninsula – Hodkinson 1983, 1986; Sabah – Cho et al. 2017), Philippines (Burckhardt and van Harten 2006, including Luzon– Hodkinson 1983), Singapore (Percy 2017), Thailand (Cho et al. 2017), Yemen (Burckhardt and Van Harten 2006).

**Host plant on Norfolk Island.***Hibiscustiliaceus* L. (Malvales, Malvaceae), confirmed by the record of immatures (Suppl. material 1).

**Remarks.** The immatures of this species congregate on the underside of leaves and in the folded new leaf growth, producing copious amounts of flocculent material (Froggatt 1901; Fig. 7E). We consider *M.hibisci* to be native to Norfolk Island based on the widespread distribution of its host plant, *Hibiscustiliaceus*, in the Pacific. However, the native status of *H.tiliaceus* on some Pacific islands has been debated as it is one of several “tramp” species in the Pacific that was used by Polynesians for medicinal purposes (Lim 2014) and therefore may have been transported between islands. Polynesians are known to have inhabited Norfolk Island 600–800 years ago but were not present at the time of European settlement (Anderson and White 2001). It can therefore not be ruled out that both host, *H.tiliaceus*, and psyllid, *M.hibisci*, were introduced to Norfolk Island by early Polynesian settlers.

#### ﻿Family Psyllidae

**Subfamily Acizzinae**

##### *Acizziaacaciaebaileyanae* (Froggatt, 1901)

Fig. 11F

*Psyllaacaciaebaileyanae* Froggatt, 1901: 257.

*Arytainaacaciaebaileyanae*; Pettey 1924: 21.

*Psyllauncata* Ferris & Klyver, 1932: 53; Tuthill 1952: 91.

*Neopsyllauncata*; Heslop-Harrison 1949: 162.

Psylla (Acizzia) acaciaebaileyanae; Tuthill 1952: 91.

*Acizziaacaciaebaileyanae*; Capener 1970: 197.

**Distribution.** Native to Australia (Froggatt 1901). Present in Africa – South Africa (Pettey 1924); Europe – Croatia (Pintar et al. 2021), France (Malausa et al. 1997), Germany (Burckhardt and Lauterer 2003), Great Britain (Malumphy and Luker 2014), Italy (Rapisarda 1985), Netherlands (Suh and Choi 2020), Poland (Seljak et al. 2004), Slovenia (Seljak 2006); Oceania – New Zealand (Ferris and Klyver 1932); Asia – Philippines (Hodkinson 1983); America – United States of America (California – Gill 1987).

**Host plant on Norfolk Island.***Acaciapodalyriifolia* A.Cunn. ex G.Don (Fabales, Fabaceae), confirmed by the record of immatures (Suppl. material 1).

**Remarks.** First report from Norfolk Island. *Acaciapodalyriifolia*, a popular garden ornamental, was not recorded as being present on the island by Green (1994) but was certainly present by 2010 (Wilcox et al. 2010), suggesting both it and *Acizziaacaciaebaileyanae* may have reached Norfolk in the intervening 16 years.

###### ﻿ Acizzia aliceae

Taxon classificationAnimaliaHemipteraAphalaridae

Percy & Martoni sp. nov.

40FB2815-B957-569A-B37D-133C264A8180

https://zoobank.org/78148BA0-595A-46F3-8664-B06C8203A406

Figs 12 , 13 , 14

####### Type locality.

Norfolk Island, Norfolk Island National Park, Summit Track, on *Dodonaeaviscosa* growing on side of the track. Collected by sweeping plant branch using a net.

####### Type material.

***Holotype*** : Norfolk Island • adult ♂; Norfolk Island National Park, Summit Track; 19 Oct. 2022; Francesco Martoni leg.; on *Dodonaeaviscosa*; sweeping; entire specimen mounted on card triangle, deposited at VAIC. Labels: “Norfolk Island N.P. / Summit Track / 19-Oct-2022 F. Martoni / On *Dodonaeaviscosa*” (Printed on white card); “HOLOTYPE ♂ / *Acizziaaliceae* / Percy & Martoni 2025” (Printed on red card). ***Paratypes***: Norfolk Island • 5 ♂♂, 5 ♀♀; same data as the holotype; dissected specimens mounted on microscope slides deposited at VAIC • 1 adult ♂, 2 adult ♀♀; same data as the holotype; entire specimens mounted on card triangle, deposited at ANIC • 2 adult ♂♂, 3 adult ♀♀; same data as the holotype; entire specimens preserved in ethanol, deposited at NHMB • 9 adult ♂♂, 4 adult ♀♀, 1 immature; Norfolk Island, 17 Rocky Point Rd; 19 Feb. 2023; James M.H Tweed leg.; on *Dodonaeaviscosa*; entire specimens preserved in ethanol, deposited at VAIC • 57 adult ♂♂, 51 adult ♀♀, 18 immatures; Norfolk Island National Park, Captain Cook Rd gate; 06 Nov. 2023; James M.H. Tweed leg.; on *Dodonaeaviscosa*; entire specimens preserved in ethanol, deposited at VAIC • 6 adult ♂♂, 2 adult ♀♀, 1 immature; Selwyn Pine Road, Highlands Ecolodge; 22 Dec. 2012; Laurence Mound leg.; on *Dodonaeaviscosa*; NI-2012-9; entire specimens preserved in ethanol, deposited at DMPC • 6 adult ♂♂, 3 adult ♀♀; Selwyn Pine Road, Highlands Ecolodge; 8 Jul. 2013; Laurence Mound leg.; on *Dodonaeaviscosa*; LAM5744; entire specimens preserved in ethanol, deposited at DMPC • 4 adult ♂♂, 3 adult ♀♀, Taylor’s Road, Burnt Pine Park; 10 Jul. 2013; Alice Wells leg.; on Dodonaeaviscosasubsp.viscosa; AW-12-83; entire specimens preserved in ethanol, deposited at DMPC • 2 adult ♂♂, 1 adult ♀; Norfolk Island Captain Cook’s Landing; 11 Jul. 2013; Alice Wells leg.; on Dodonaeaviscosasubsp.viscosa; AW-12-90; entire specimens preserved in ethanol, deposited at DMPC • 13 adult ♂♂, 9 adult ♀♀; J.E. Road, Highlands Lodge; 22 Oct. 2013; Alice Wells leg.; on *Dodonaeaviscosa*; AW-008; entire specimens preserved in ethanol, deposited at DMPC. All paratypes are labelled as “PARATYPE ♂-♀ / *Acizziaaliceae* / Percy & Martoni 2025” (Printed on blue card).

####### Diagnosis.

Although a number of undescribed species of *Acizzia* associated with *Dodonaea* have been reported recently, and are in the process of being described (McClelland et al. 2025), the only currently described species of *Acizzia* associated with *Dodonaea* is *Acizziadodonaeae* (Tuthill, 1952). Geographically, distributions of *A.aliceae* and *A.dodonaeae* do not overlap, and the two species are easily distinguished morphologically due to the absence of any wing pattern in *A.aliceae.* Furthermore, the male parameres of *A.aliceae* are broader and less sinuous than those of *A.dodonaeae*, while the female proctiger is less arched posteriorly (Tuthill 1952).

####### Description.

**Figures 12.**
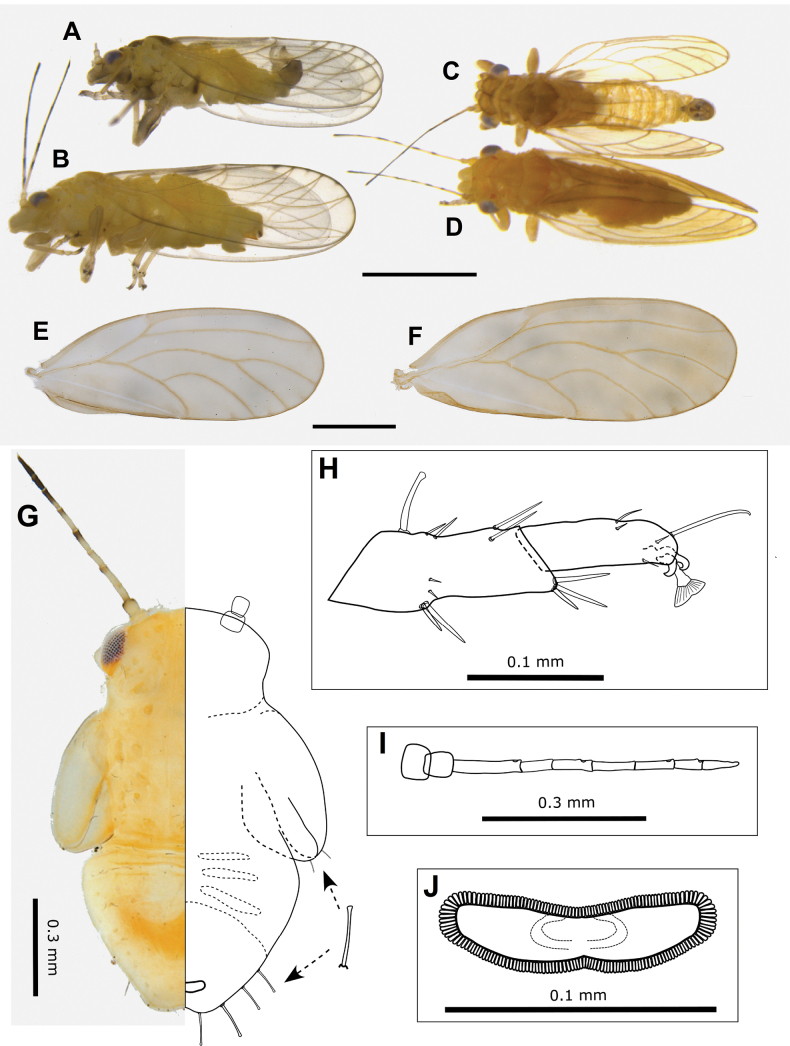
Adults of *Acizziaaliceae* Percy & Martoni, sp. nov. **A** adult lateral habitus of male **B** same, female **C** dorsal habitus of male **D** same, female **E** fore wing of male **F** same, female **G** immature habitus showing placement of long capitate setae on abdomen and wing pads **H** metatibiotarsus of immature **I** antenna of immature **J** circumanal ring of immature. Scale bars: 1 mm (**A–D**); 0.5 mm (**E, F**); 0.3 mm (**G, I**); 0.1 mm (**H, J**).

***Colouration*.** Adult. Body generally pale to dark green (dried or ethanol-preserved material yellow); dorsum of thorax varying from a dark green to a dark brown (Fig. 12A–D), pronotum, mesopraescutum and mesoscutum all with longitudinal parallel darker bands. Abdomen lighter coloured. Antennal segments 1 and 2 lighter, segments 3–7 darkening apically, segments 8–10 uniformly dark (Fig. 12B–D). Wings hyaline, often yellowish, with darker veins and radular areas (Fig. 12E, F). Male terminalia pale, with paramere tips darker (Fig. 12A, C). Female terminalia pale, with tip of proctiger darker (Fig. 12B, D).

Immature (5^th^ instar): Body pale green to yellow-green (Fig. 12G). First four segments of antenna uniformly yellow-green, segments 5, 6, and 7 tending to a darker colour in the apical portion, last three segments of antennae uniformly dark brown-black.

***Structure*.** Adult. Body relatively large, 2–3 mm from anterior margin of vertex to tip of folded wings (males smaller than females) (Fig. 12A–D).

**Figure 13.**
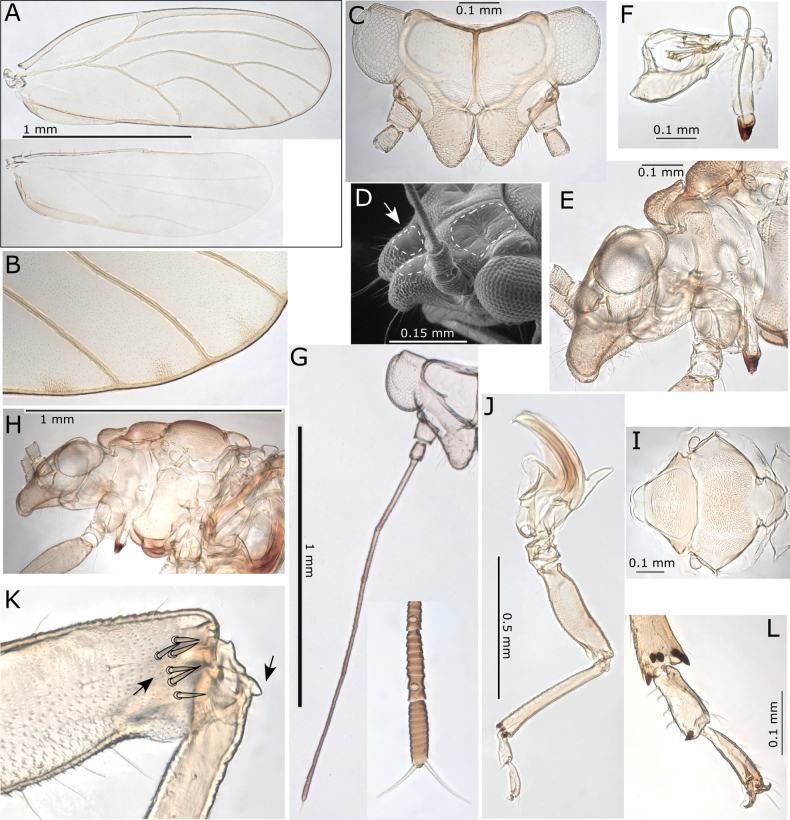
*Acizziaaliceae* Percy & Martoni, sp. nov. **A** fore wing and hind wing (below) **B** detail of radular spinule cluster positions **C** head (dorsal view) **D** head (dorso-lateral view) indicating naked patches at base of genal cones and around discal foveae **E** head (ventro-lateral) showing genal cones downturned apically **F** clypeus and proboscis **G** antennae with apical detail (inset) **H** head and thorax (lateral view) **I** thorax (dorsal view) **J** hind leg **K** metafemur apex indicating cluster of stout lateral setae and genual spine on metatibia **L** metatarsi showing a single outer metabasitarsus spine. Scale bars as reported.

Head (Fig. 13C–G) in lateral view slightly deflexed downward, wider than thorax, width < 0.5 × antennal length, vertex width almost 2 × length, with well-defined cranial suture. Genal processes well developed, conical, diverging and distinctly downturned at apices, length ~ 3/4 vertex length, apices rounded, curiously naked patches on the dorsal surface at base lacking setae or microsculpture, similarly naked areas surround the discal foveae on vertex (Fig. 13D). Clypeus subglobular, terminal proboscis segment short (Fig. 13F). Antennae 10-segmented, with segment 3 longest, length of segments 4–8 subequal, and the shorter terminal segments 9 and 10 subequal; a single subapical rhinarium on segments 4, 6, 8, 9, simple, circular, length of two long terminal setae subequal, with both shorter than segment 10 (Fig. 13G).

Thorax (Fig. 13H, I) moderately arched in lateral profile. Hind legs (Fig. 13J–L) with meracanthus well developed, thorn-like and slightly curved; metafemur shorter than metatibia and with a cluster of 4–6 stout setae subapically on outer lateral surface (Fig. 13L); metatibia with a single genual spine basally and 1+4 (typically with two close together) or occasionally 1+3 sclerotised apical spurs surrounded by a crown of 8–10 stout setae; length of metatarsal segments subequal; metabasitarsus with a single outer sclerotised spur.

Fore wing (Figs 12E, F, 13A) length > 2.5 × width, more or less parallel sided, widest in apical half, rounded at apex; pterostigma long and slender; cells m_1_ and cu_1_ both elongate, but m_1_ narrower and more elongate than cu_1_, vein Rs long and moderately sinuous, vein M much shorter than Rs and strongly arched; membrane with spinules densely distributed in all cells and with spinule-free bands along veins, marginal radular spinule clusters positioned centrally in cells m_1_ and m_2_, and in posterior half of cell cu_1_ (Fig. 13B).

**Figure 14.**
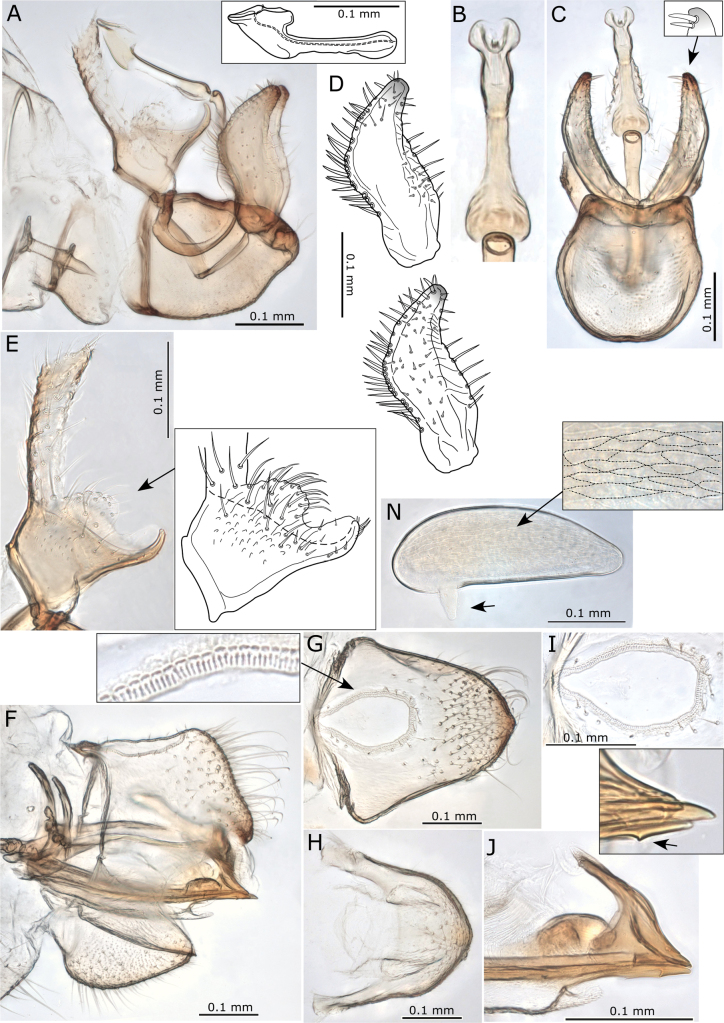
*Acizziaaliceae* Percy & Martoni, sp. nov. **A** male terminalia with aedeagus detail (inset) **B** aedeagus (posterior view) **C** male terminalia (posterior view) with detail of paramere apex (inset) **D** paramere outer surface (above) and inner surface (below) **E** male proctiger (lateral view) with detail of basal portion (illustrated) **F** female terminalia (lateral view) **G** female proctiger (dorsal view) with detail of anal ring pores **H** female subgenital plate (ventral view) **I** anal ring (dorsal view) **J** ovipositor (lateral view) with detail of single subapical tooth on valvulae ventralis (inset). Scale bars as reported.

Male terminalia (Fig. 14A–D) with subgenital plate somewhat elongate, length greater than height, dorsal margin sinuate and ventral margin not evenly rounded (Fig. 14A, C). Proctiger shorter than paramere, expanded basally into distinct basal posterior lobes that extend outwards from below and around a weakly sclerotised hook-like appendage, upper portion narrow, cylindrical, but tubular only in apical 1/3 (Fig. 14E). Paramere in lateral profile, sinuous, widest in middle, anterior margin medially arched forward and bearing many long setae, posterior margin moderately concave, bearing fewer long setae, apex blunt and directed rearward, moderately sclerotised and bearing two distinctly stout setae subapically on inner surface, otherwise inner surface with scattered short setae, outer surface with few short setae concentrated towards the posterior margin (Fig. 14A, C, D). Distal segment of aedeagus moderately long, base expanded laterally, apical portion somewhat saccate below a bluntly acute tip and deeply incised dorsum, apical portion ~ 1/3 as long as the distal segment length (Fig. 14B, D).

Female terminalia (Fig. 14F–J) short, with proctiger arched dorsally and steeply downturned post anus, covered in numerous long setae in the distal half and terminating in an acute, sclerotised tip (Fig. 14F), in dorsal view broadly rounded apically; anal ring outline in dorsal view pyriform, narrower anteriorly and broader posteriorly, length ~ 1/2 proctiger length, composed of a double row of regular pores (Fig. 14G, I); subgenital plate shorter than proctiger, length ~ 3/4 proctiger length, apex blunt except for a small beak and whole covered in medium to short setae (Fig. 14F, H); ovipositor dorsal valvulae triangular but with a long narrow extension dorsally, ventral valvulae bearing a single small tooth ventrally (Fig. 14J). Egg elongate with “puzzle-piece” striations over most of the surface, and a short, stout, lateral pedicel 1/4 length from base, apical filament apparently lacking (Fig. 14N).

Immature (5^th^ instar): body ~ 1.55 × as long as wide, shape ovoid, wing pads protruding (Fig. 12G). Setae predominantly a mix of medium to long simple and weakly capitate, with a single long capitate seta at the apex of each wing pad, a few shorter capitate setae on antennae and legs, and abdomen with 4+4 long capitate setae (Fig. 12G). Antenna length ~ 1.3 × head width, 9-segmented with a single subapical rhinarium on segments 3, 5, 7, and 8, segment 3 longest and approx. equal to length of segments 8 and 9 combined (Fig. 12G, I). Fore wing pads lacking humeral lobes. Tarsal arolium longer than claws, triangular with unguitractor and long pedicel (Fig. 12H), claws well developed. Anus in ventral position (Fig. 12G), circumanal ring shallowly heart-shaped, consisting of a single row of pores (Fig. 12J).

***Measurements*** (in mm). Adults (5 ♂♂, 5 ♀♀). Length of body (vertex to terminalia) ♂ 1.66–1.93, ♀ 2.07–2.32; length of body (vertex to apex of folded wings) ♂ 2.33–2.46, ♀ 2.79–2.94; width of head (HW) ♂ 0.56–0.59, ♀ 0.64–0.68; length of genal processes (GCL) ♂ 0.11–0.13, ♀ 0.14–0.17; length of vertex (VL) ♂ 0.15–0.17, ♀ 0.18–0.19; width of vertex (VW) ♂ 0.32–0.36, ♀ 0.39–0.40; length of antenna (AL) ♂ 1.34–1.38, ♀ 1.30–1.38; length of fore wing (WL) ♂ 1.54–1.81, ♀ 2.15–2.19; width of fore wing ♂ 0.59–0.71, ♀ 0.80–0.83; length of hind wing ♂ 1.43–1.66, ♀ 1.80–1.87; length of vein Rs ♂ 1.05–1.13, ♀ 1.36–1.41; length of vein M(M) ♂ 0.59–0.72, ♀ 0.78–0.86; length of vein M_1+2_ (M1) ♂ 0.51–0.56, ♀ 0.67–0.75; marginal width of cell m_1_ ♂ 0.22–0.24, ♀ 0.26–0.30; marginal width of cell cu_1_ ♂ 0.35–0.44, ♀ 0.52–0.54; length of vein Cu_1b_ ♂ 0.25–0.30, ♀ 0.30–0.36; value of cell cu_1_ ♂ 1.35–1.70, ♀ 1.64–1.89; value of cell m_1_ ♂ 2.18–2.54, ♀ 2.40–2.67; length (height) of proctiger (PL) ♂ 0.14–0.19; I length of paramere ♂ 0.18–0.22; length of distal aedeagal segment ♂ 0.17–0.20; length of subgenital plate ♂ 0.23; height of subgenital plate ♂ 0.17–0.19; length of proctiger (PL) ♀ 0.28–0.31; length of circum-anal ring (CL) ♂ 0.13–0.15; length of subgenital plate (SL) ♀ 0.24–0.28. Ratios: GCL:VL ♂ 0.65–0.81, ♀ 0.78–0.94; VL:VW ♂ 0.42–0.53, ♀ 0.45–0.49; VL:HW ♂ 0.26–0.30, ♀ 0.27–0.29; AL:HW ♂ 2.34–2.39, ♀ 2.03; PL:HW ♂ 0.25–0.41, ♀ 0.42–0.48; PL:CL ♀ 1.93–2.23; PL:SL ♀ 1.07–1.17; WL:HW ♂ 2.65–3.12, ♀ 3.21–3.37; WL:WW ♂ 2.44–2.95, ♀ 2.63–2.74; Rs:M ♂ 1.53–1.78, ♀ 1.60–1.74; M1:M ♂ 0.72–0.95, ♀ 0.78–0.94.

Immatures (5^th^ instar, 1 specimen). Length of body 1.43; width of body 0.92; length of antennae 0.76; width of head 0.57.

####### Etymology.

The species epithet was chosen to honour the Australian entomologist, Dr Alice Wells, for her fundamental contribution to entomology in the Austro-Pacific. Dr Wells was likely the first person to collect this species during the 2012–2014 survey (Maynard et al. 2018).

####### Distribution.

**Figure 15.**
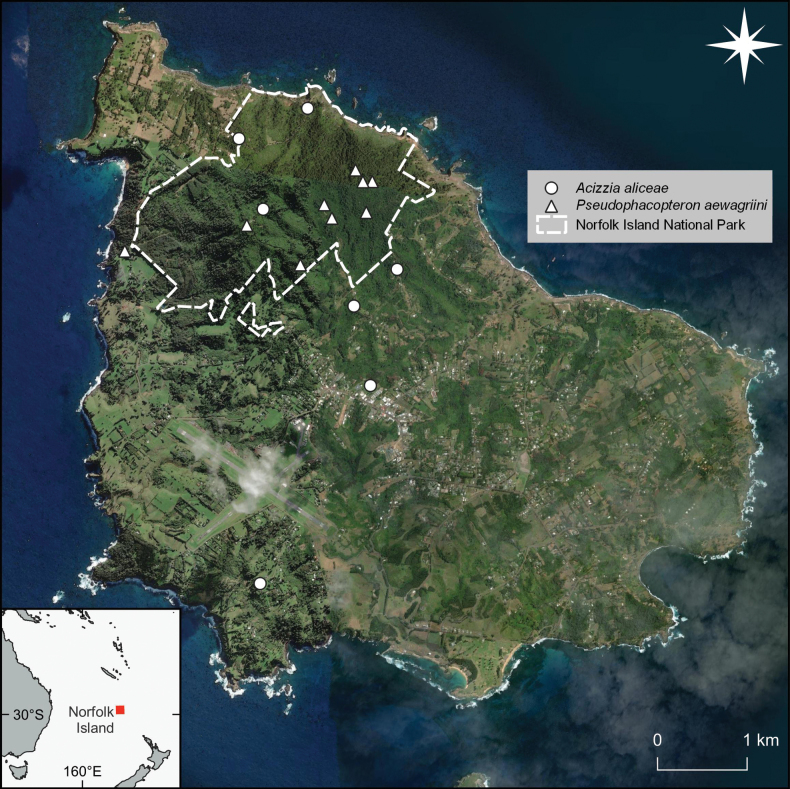
Confirmed occurrence records for *Pseudophacopteronaewagriini* Percy & Martoni, sp. nov. (triangles) and *Acizziaaliceae* Percy & Martoni, sp. nov. (circles). The dotted line indicates the boundaries of Norfolk Island National Park and Botanic Gardens. Records shown are derived from both collected material and confirmed records from iNaturalist (https://www.inaturalist.org/) and personal communications. Map constructed in QGIS with satellite imagery sourced from ESRI World Imagery.

This species is widely distributed on Norfolk Island and is likely present anywhere the host plant is found (Fig. 15).

####### Host plant on Norfolk Island.

*Dodonaeaviscosa* Jacq. (Sapindales, Sapindaceae), confirmed by the collection of immatures (Suppl. material 1).

####### Conservation.

This species is considered endemic to Norfolk Island. It specialises on a native host plant, *Dodonaeaviscosa*, which is widespread in tropical, subtropical, and warm temperate regions around the world. During this study, *Acizziaaliceae* was found to be present within all stands of *Dodonaeaviscosa* searched on the island, including natural populations and specimens planted ornamentally. It is assumed that this species is widespread across the island and occurs, or can occur, anywhere the host plant is present. Neither species is known from nearby Phillip Island. The EOO ranges from 5.5–42.2 km^2^ depending on whether this is calculated based on confirmed occurrence records, or the entire area of Norfolk Island (Fig. 15). The corresponding AOO calculated using a 4 km^2^ grid overlay, ranges from 16–64 km^2^. There are no known threats facing this species, nor is it known to be undergoing a population or range decline.

We propose that *A.aliceae* warrants a threat status of Least Concern (IUCN, 2012). Its widespread distribution on the island, including within both protected areas and urban and agricultural landscapes, as well as on both natural and planted populations of *D.viscosa* suggests it is tolerant of a range of conditions and capable of dispersing between host plant patches. The adaptability of its host plant and its widespread use in garden plantings and hedgerows, suggests its habitat is secure and has likely increased in extent since European colonisation of the island. Despite its relatively small EOO and AOO, there are no known ongoing or potential future threats and so this species does not qualify for any of the threatened categories under any of the criteria (IUCN 2012). However, encouraging planting of *D.viscosa* on public and private land, as well as on nearby Phillip Island, would further secure this species.

####### DNA resources.

GenBank COI: MG988625, cytB: MG988895. Also represented in the mitogenome analysis of Percy et al. (2018) as: DP2.idba.202. The annotated mitochondrial genome was submitted to GenBank for this study (PQ754209). Additionally, a total of four COI sequences were generated for this study (OR558301, OR558302; OR558308, OR558309).

####### Systematics.

**Figure 16.**
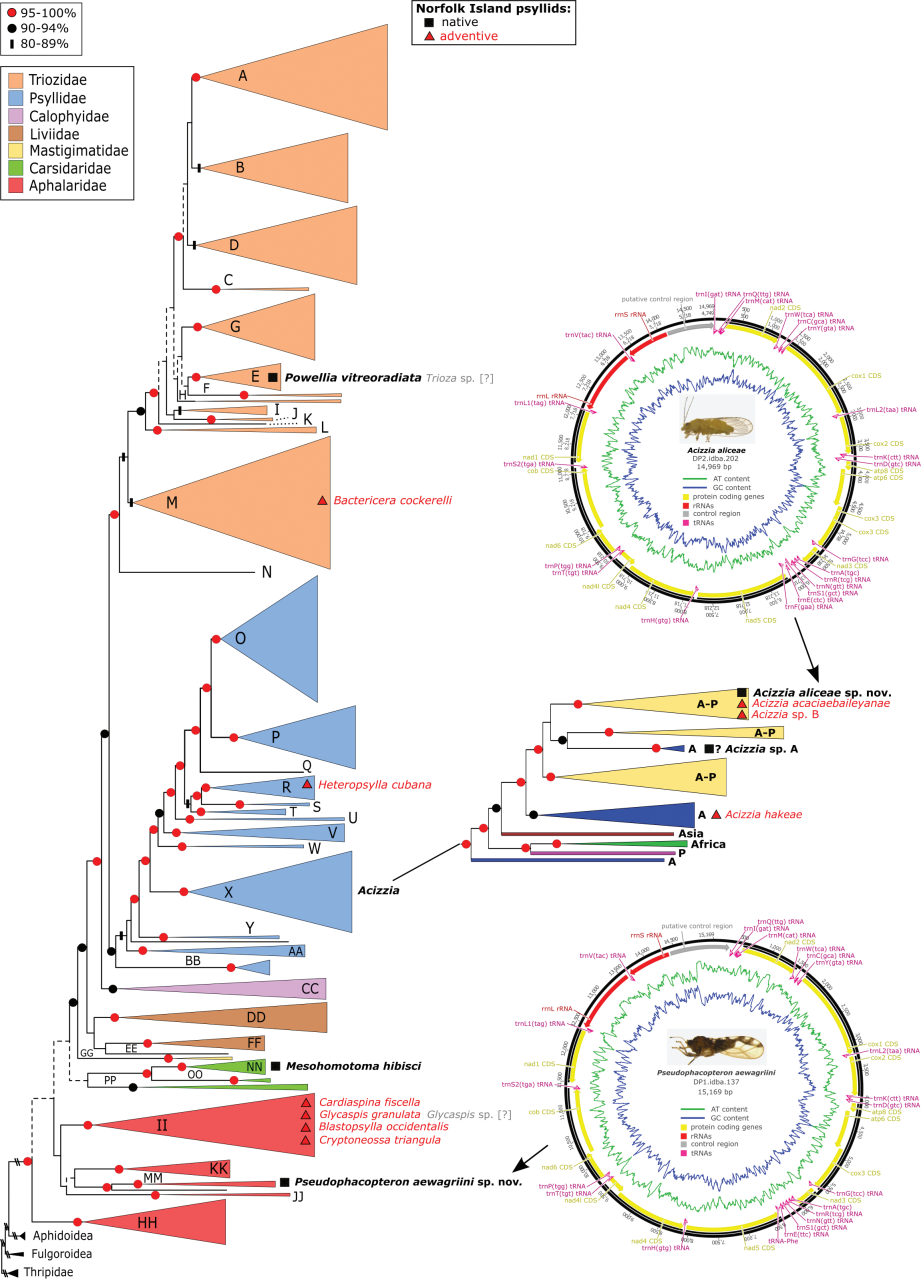
Systematic placement of the Norfolk Island psyllid fauna (native taxa in black bold, adventive taxa in red) determined using a maximum likelihood backbone constraint analysis with the mitogenome data from Percy et al. (2018). The two annotated genomes for the endemic species, *Acizziaaliceae* Percy & Martoni, sp. nov. and *Pseudophacopteronaewagriini* Percy & Martoni, sp. nov. are illustrated as well as more detailed clade placement for *Acizzia* spp. (A – Australia, P – Pacific, A-P – Austro-Pacific). Two taxa indicated by [? in grey] are referred to as *Glycaspis* sp. and *Trioza* sp. in Maynard et al. (2018) and are considered here to refer to *Glycaspisgranulata* and *Powelliavitreoradiata*, respectively (see Discussion).

Related to *Acizziadodonaeae* (Tuthill, 1952) from New Zealand, and both species are in the same subgroup of *Acizzia* that includes *A.uncatoides* and *A.acaciaebaileyanae* (Percy et al. 2018), as well as all but two of the Norfolk Island *Acizzia* reported here (Fig. 16).

##### *Acizziahakeae* (Tuthill, 1952)

Fig. 11G

Psylla (Acizzia) hakeae Tuthill, 1952: 91.

*Acizziahakeae*; Loginova 1977: 577.

**Distribution.** This species was described from New Zealand and hypothesised to be originally from Australia based on host plant association (Tuthill 1952; see remarks). Also recorded in the USA (Percy et al. 2012).

**Host plant on Norfolk Island.** Unknown from Norfolk Island (see Remarks).

**Remarks.** This is the first report of this species from Norfolk Island, based on a single adult female collected using a Malaise trap set in rainforest within Norfolk Island National Park. This species has been recorded from New Zealand and USA on *Hakea* and *Grevillea* (Proteaceae), popular garden ornamentals. The only plant species reported from Norfolk Island for these genera are *Hakeasericea* Schrad., *Hakeasalicifolia* (Vent.) B.L.Burtt, and *Grevillearobusta* A.Cunn. ex R.Br (Green 1994). Therefore, these are hypothesised here to be the host for *A.hakeae* on Norfolk Island.

##### *Acizzia* sp. A

Fig. 11H

**Distribution.** First report from Norfolk Island. Additional distribution is unknown (see Remarks).

**Host plant on Norfolk Island.** Collected from *Dodonaeaviscosa*, although this may not be the host plant (see Remarks).

**Remarks.** Possibly native or endemic to Norfolk Island, since no records of this species from elsewhere could be confirmed. Only a single female individual was collected, suggesting the host plant may be a different species and the presence on *D.viscosa* may only be incidental (e.g., windblown). *Acizzia* species are often associated with *Acacia*, particularly in Australia (Ouvrard 2020, Martoni et al. 2024), and *Acacia* may be a host plant for this species. However, only a single female specimen was collected despite extensive searches of the surrounding area, and no *Acacia* plants were in the vicinity. As such, the status of *Acizzia* sp. A cannot yet be confirmed, with further survey effort needed to confirm host plant and distribution on Norfolk Island. Interestingly, the DNA analysis places this taxon outside the Austro-Pacific clade containing the other *Acizzia* from Norfolk Island, separate from *Acizziahakeae*, and in a smaller clade of exclusively Australian taxa (Fig. 16); the caveat is that the molecular sampling represents only a small part of this speciose genus. The COI sequence generated in this study is the only sequence for this taxon that is currently available.

##### *Acizzia* sp. B

Fig. 11I

**Distribution.** Present also in Australia (Queensland; Martoni et al. 2020).

**Host plant on Norfolk Island.***Acaciaspirorbis* Labill. (Fabales, Fabaceae), confirmed by the collection of immatures (Suppl. material 1).

**Remarks.** First report from Norfolk Island. A number of specimens (40 males, 46 females, 27 immatures; Suppl. material 1) were collected from a very old *A.spirorbis* specimen planted near the centre of the Burnt Pine township, seemingly one of the very few *A.spirorbis* plants present on the island. The same species was recorded in Queensland in 2020 using light trapping (Martoni et al. 2020, as “*Acizzia* sp. B”). This previous Australian record, together with the association with an introduced host plant which is native to Australia, as well as New Caledonia and other Pacific Islands, suggest this psyllid species is adventive to Norfolk Island. *Acizzia* sp. B is probably an undescribed species, since there are no previous reports of *A.spirorbis* as a host plant (Hollis 2004; Martoni et al. 2024). The DNA analysis places this taxon in the same Austro-Pacific clade containing most of the other *Acizzia* from Norfolk Island (Fig. 16).

#### ﻿Subfamily Ciriacreminae

##### *Heteropsyllacubana* Crawford, 1914

Fig. 11J

*Rhinocolaincisa* Šulc, 1914; synonymised in Burckhardt 1986: 1023.

*Heteropsyllaincisa*; Tuthill 1959: 13.

**Distribution.** First official report from Norfolk Island. Originally described from Cuba (Crawford 1914). Adventive to Australia (Bray and Sands 1987). Present in America – Bahamas (Brown and Hodkinson 1988), Bermuda (Muddiman et al. 1992), Brazil (Burckhardt and Queiroz 2012), Chile (Olivares and Burckhardt 2002), Colombia (Hodkinson 1988), Costa Rica (Muddiman et al. 1992), Cuba (Crawford 1914), Dominican Republic (Muddiman et al. 1992), Ecuador (Hodkinson and Muddiman 1993), El Salvador (Brown and Hodkinson 1988), Guatemala, Jamaica, Mexico (Muddiman et al. 1992), Nicaragua, Panama (Brown and Hodkinson 1988), Peru, Suriname (Hodkinson and White 1981), Trinidad-Tobago (Muddiman et al. 1992), United States of America (California – Percy et al. 2012; Florida – Hodkinson 1988; Puerto Rico – Hodkinson and White 1981; Virgin Islands – Brown and Hodkinson 1988); Asia – Bangladesh (Burckhardt et al. 2018), Cambodia (Napompeth 1994), China (Hong Kong – Martin and Lau 2011), Haiti, India, Indonesia (Muddiman et al. 1992), Japan (Bonin and Ryukyu Islands – Inoue and Miyatake 2001), Laos (Cho et al. 2017), Malaysia (Muddiman et al. 1992), Nepal, Philippines, Sri Lanka, Taiwan, Thailand, (Muddiman et al. 1992), Vietnam (Geiger and Gutierrez 2000); Africa – Burundi (Napompeth 1994), Cameroon (Dzokou et al. 2009), Reunion Island, Kenya, Mauritius (Muddiman et al. 1992), South Africa (Olckers 2011), Tanzania, Uganda (Napompeth 1994), Zimbabwe (Matimati et al. 2009); Oceania – Christmas Island, Cook Islands, Fiji, New Caledonia (Muddiman et al. 1992), French Polynesia, Austral Islands (Tubuai Islands) (Claridge et al. 2014), Mariana islands, Niue, Papua New Guinea, Samoa, Solomon Islands, Tonga (Muddiman et al. 1992), USA (Guam, Hawaii – Muddiman et al. 1992).

**Host plant on Norfolk Island.***Leucaenaleucocephala* (Lam.) de Wit (Fabales, Fabaceae), confirmed by the collection of immatures (Suppl. material 1).

**Remarks.** Adventive to Norfolk Island. First record of this species.

#### ﻿Family Triozidae

##### *Bactericeracockerelli* (Šulc, 1909)

Fig. 11K

*Triozacockerelli* Šulc, 1909: 102.

*Paratriozacockerelli*; Crawford 1911: 448.

*Bactericeracockerelli*; Burckhardt and Lauterer 1997: 124.

**Distribution.** Originally described from the United States of America (Šulc 1909) and considered native to Western North America and introduced everywhere else. Previously reported on Norfolk Island (Walker et al. 2015). Present in Australia (Western Australia – PHA 2019), Canada (Alberta, British Columbia, Saskatchewan – Hodkinson 1988; Ontario – Butler and Trumble 2012), Ecuador (Castillo Carrillo et al. 2019), El Salvador (EPPO 2013), Guatemala (Powell et al. 2012), Honduras (Butler and Trumble 2012), Mexico (Hodkinson and White 1981), New Zealand (Teulon et al. 2009), Nicaragua (EPPO 2013), United States of America (Arizona, California, Idaho, Iowa, Kansas, Minnesota, Montana, Nevada, New Mexico, North Dakota, Oklahoma, South Dakota, Utah – Hodkinson 1988; Colorado – Šulc 1909; Nebraska – Wallis 1946; Oregon – Butler and Trumble 2012; Texas – Janes 1939; Washington – Butler and Trumble 2012; Wyoming – Wallis 1946); it has been intercepted in Florida but not considered established there (Halbert and Burckhardt 2020).

**Host plants on Norfolk Island.***Solanumlycopersicum* L. (Solanales, Solanaceae) and *Capsicum* sp. (Solanales, Solanaceae). No immatures were collected (Suppl. material 1), but leaf curling was observed.

##### *Powelliavitreoradiata* Maskell, 1879

Fig. 11L

*Triozapellucida* Maskell, 1890: 164; synonymised in Ferris and Klyver 1932: 36.

**Distribution.** Originally described from New Zealand (Maskell 1879) and previously reported on Norfolk Island (Maynard et al. 2018). Present in Europe – France (Cocquempot 2008), Great Britain (Martin and Malumphy 1995), Ireland (O’Connor et al. 2004).

**Host plants on Norfolk Island.***Pittosporumbracteolatum* Endl. and *P.undulatum* Vent. (Apiales, Pittosporaceae). Immatures and pitting on the leaves were observed on both plant species, but only two immatures were collected on *P.undulatum* (Suppl. material 1).

**Remarks.** This species is considered to be native to Norfolk Island, since one of the host plants is endemic to the archipelago. While the distribution of this psyllid across New Zealand (where it is native) and Europe (where it is adventive) makes it a widespread taxon, its distribution on Norfolk Island is constrained by the distribution of *P.bracteolatum*, which is not only endemic but also classified as “vulnerable” (Coyne 2019), and possibly *P.undulatum.* Neither of the Norfolk Island hosts are known from New Zealand, where the psyllid is native. *Pittosporumundulatum* is an Australian mainland introduction to Norfolk Island, suggesting that the host breadth of *Powelliavitreoradiata* likely expanded after *P.undulatum* was introduced, as has occurred in other regions (Salisbury et al. 2011). Although the shallow pit galls made on the leaves can cause leaf distortion and chlorosis (Fig. 7F), and in high densities this psyllid has been considered a significant pest to the horticultural trade (Mifsud et al. 2010), there is no evidence at this time that *P.vitreoradiata* is detrimental to endemic *P.bracteolatum*.

A note on polymorphism in immatures: first described by Carter (1949) and more recently by Martin (2010), immatures of 1^st^ and 2^nd^ instars always have dorsal sectasetae (similar in type to the marginal sectasetae), but in 3^rd^ to 5^th^ instars these dorsal setae can remain, or are few, or are lost completely. Immature colour also varies considerably, from pale yellow or green to dark brown. Carter (1949) considered the presence of darker colouration and absence of dorsal setae to indicate habitation on the upper, sun exposed, glabrous leaf surface, and paler immatures with dorsal setae to be found generally on the lower leaf surface. However, Martin (2010) found no such correlation. Only two 5^th^ instar immatures were examined from Norfolk Island and both had dorsal setae present, while another collection examined from New Zealand had only “naked” 5^th^ instars, and material examined from an adventive population from the United Kingdom had both types (Suppl. material 1).

### ﻿Systematics of the Norfolk Island Psylloidea

Fig. 16 illustrates the phylogenetic position of the Norfolk Island taxa, with native species in four families: Aphalaridae, Carsidaridae, Psyllidae, and Triozidae. The systematic placement of the Norfolk Island taxa within the Psylloidea phylogeny is mostly well resolved and supported. This is in part due to all of the genera and seven of the species that occur on Norfolk Island already represented in the original mitogenome data (Percy et al. 2018). However, the more detailed clade placement illustrated in Fig. 16 of some of the *Acizzia* species not originally included in the mitogenome data are placed with short sequences only, and these should be considered best estimates until further data can allow a fully resolved placement within *Acizzia*.

## ﻿Discussion

Only a handful of insect groups on Norfolk Island have received comprehensive attention, with notable examples including the Lepidoptera (Holloway 1977), Carabidae (Coleoptera) (Moore 1985), Orthoptera (Otte and Rentz 1985; Rentz 1988), and most recently, Thysanoptera (Mound and Wells 2015). This study goes some way to filling that gap for the Psylloidea.

In this study, we report a total of 14 psyllid species for Norfolk Island, eight of which are reported here for the first time. We consider that two of the taxa only identified to genus in Maynard et al. (2018), namely *Trioza* sp. and *Glycaspis* sp. belong to *Powelliavitreoradiata* and *Glycaspisgranulata*, respectively. *Powelliavitreoradiata* was reascribed from *Trioza* to *Powellia* in the revision of Burckhardt and Ouvrard (2012), a genus that now includes most of the New Zealand endemic triozids (Martoni et al. 2021).

Of the 14 species recorded, two species are endemic, two are considered native, one has an unknown distribution, and nine are considered adventive. Four of the nine adventive species are Spondyliaspidinae (Aphalaridae) associated with *Eucalyptus*. The timing of introduction and selection of particular *Eucalyptus* species for plantations on the island has determined this element of the fauna. In Australia, there are more than 240 described eucalypt-feeding aphalarids (Martoni et al. 2024), and four of these are known from Norfolk Island. At least nine eucalypts have been recorded on Norfolk Island (Wilcox et al. 2010), but *Eucalyptusbotryoides* and *Eucalyptusmicrocorys* are the species most commonly planted. While *Cardiaspinafiscella* and *Glycaspisgranulata* have also been recorded from *E.botryoides* in Australia, the only record of *Blastopsyllaoccidentalis* from this host is from Norfolk Island (Maynard et al. 2018). A similar situation likely applies to the origin of the two species of *Acacia*-feeding *Acizzia*: *A.acaciaebaileyanae* and *Acizzia* sp. B. No species of *Acacia* are native to Norfolk Island; therefore, both species are likely to be adventive along with their respective host plants. The precise host of *A.hakeae* on Norfolk Island remains to be confirmed as both *Hakea* and *Grevillea* species are known to be present on the island (Green 1994), however, as the single specimen was captured using a Malaise trap, this can not yet be confirmed. Finally, *Bactericeracockerelli* and *Heteropsyllacubana*, are known to be widespread worldwide where they are commonly associated with the same host plants reported for Norfolk Island (Ouvrard 2020).

This study provides the first confirmation of endemic psyllids on Norfolk Island. *Pseudophacopteronaewagriini* Percy & Martoni, sp. nov. and *Acizziaaliceae* Percy & Martoni, sp. nov. likely both have Australasian ancestral origins. However, while the host plant of *P.aewagriini*, *Alyxiagynopogon*, is endemic to Norfolk Island, the host plant of *A.aliceae*, *Dodonaeaviscosa*, is a widespread species naturally occurring pantropically (Harrington and Gadek 2009). Al­though *Acizziaaliceae* is only the second species of *Acizzia* described from *Dodonaea*, a recent study has highlighted the presence of another eight species associated with this plant genus from Australia (McClelland et al. 2025).

Finally, the species *Mesohomotomahibisci* and *Powelliavitreoradiata* are considered native to Norfolk Island due to their host plant range. *Hibiscustiliaceus* (host of *M.hibisci*) and *Pittosporumbracteolatum* (host of *P.vitreoradiata*) are native and endemic to Norfolk Island, respectively; adult *P.vitreoradiata* have also been recorded from introduced *Pittosporumundulatum* on Norfolk Island. Additionally, the native status of *H.tiliaceus* on some Pacific islands has been debated (Lim 2014) and it is not entirely certain that it was not introduced on Norfolk Island prior to European settlement. In summary, although treated here as native, further research and large-scale population studies on these more widespread Pacific taxa would be needed to assess the questions of native versus adventive.

In general, the psyllid fauna composition on Norfolk Island has similar elements to that of nearby islands (e.g., New Zealand, New Caledonia, Cook Islands – Martoni et al. 2016; Martoni and Brown 2018; Ouvrard 2020), with a mix of endemic, native, and adventive species, often shaped and modified by the introduction of plants and associated insects. While most of the adventive species are native to Australia, these are also present in New Zealand, suggesting that most of the introductions may have originated from there. Given the small size of Norfolk Island and the large-scale clearing of indigenous vegetation, it is possible that other native psyllid species may have been extirpated before being recorded. Similarly, despite the authors’ sampling efforts, it is possible that other species await discovery, particularly given many of Norfolk Island’s indigenous plant species, or their congeners, have known psyllid associations in other areas of their range. Furthermore, there has clearly been a proliferation of adventive psyllid species on economically important introduced hosts (e.g., eucalypts and solanaceous crops). One Norfolk Island invasive psyllid, *Heteropsyllacubana*, is widely known as a pest worldwide due to its association with *Leucaena* where this plant is valued as a forage crop. *Leucaena* was introduced to Norfolk Island, possibly by mistake due to its similarity with ornamental *Albizia* species and lack of local value, and therefore the role of the psyllid may be more as a biological control agent of *Leucaena* (which can be an invasive weed), although its effectiveness in this role has been questioned (Olckers 2011).

Interestingly, some of the species present elsewhere show different ecological traits on Norfolk Island. For example, the apparently native *Powelliavitreoradiata* feeds on the endemic plant *Pittosporumbracteolatum*, but was also found on introduced *P.undulatum* (as it is in other regions where both psyllid and plant have been introduced; Salisbury et al. 2011), suggesting that, if this species is native as we propose, it has expanded its host range on the island to include introduced *Pittosporum*; it is also known to be oligophagous on several different *Pittosporum* species in other regions. *Pittosporumbracteolatum* is not only endemic to Norfolk Island but classified as “vulnerable”, with only 921 plants known in 2003 (Coyne 2019), although intensive planting has now been conducted as part of its management plan (Director of National park 2010; Commonwealth of Australia 2025). The impact of this psyllid on the host may require further assessment, but based on field observations conducted during this study it is considered highly unlikely that growth of this endemic plant is strongly impacted by the insect. Preservation of this vulnerable plant on Norfolk Island will also be important to preserve the local psyllid population.

The backbone constraint tree method has proven to be a reliable option to obtain effective best estimate systematic placements of taxa with limited sequence data, particularly with increasing genome data available to construct backbone frameworks (Boyle and Adamowicz 2015; Percy et al. 2018; Macías-Hernández et al. 2020; Jiménez-García et al. 2023; Bastin et al. 2024). With regard to the systematic placement of the Norfolk Island taxa, all the genera found on Norfolk Island were already represented in the mitogenome data of Percy et al. (2018) providing relatively accurate systematic placement of new sequence data. Interestingly, most of the *Acizzia* on Norfolk Island, both native and non-native, belong to the same clade of mixed Austro-Pacific (mixed Australian and Pacific species), the exception are *Acizzia* sp. A and *Acizziahakeae*, which were recovered in two separate clades of exclusively Australian taxa (Fig. 16).

The discovery of endemic psyllids on Norfolk Island, which has been heavily modified since European colonisation in 1788, highlights the importance of protected areas such as National Parks and Reserves. Many of Norfolk Island’s described endemic insect species have the majority, if not all (e.g., *Pseudophacopteronaewagriini* Percy & Martoni, sp. nov.), of their known occurrence records within the only National Park of the island, highlighting its importance for the conservation of both described and as yet undiscovered endemic insect species.

## ﻿Conclusions

This study updates our general understanding of the psyllid fauna of Norfolk Island, adding seven more species to those previously reported, including the first report of endemic psyllids for the archipelago, and providing an identification key to the species. The presence of endemic species, *Pseudophacopteronaewagriini* Percy & Martoni, sp. nov. and *Acizziaaliceae* Percy & Martoni, sp. nov., highlights the importance of protected areas such as National Parks and Reserves, even in areas of the world that have been heavily modified by human impact. Ultimately, this work generates important information for the superfamily Psylloidea, which is often not well characterised in faunistic surveys, improving our understanding of the biodiversity of this group in the South Pacific.

## Supplementary Material

XML Treatment for Pseudophacopteron aewagriini

XML Treatment for Acizzia aliceae

## Footnotes

note: at least two other Acizzia species also occur on Acacia in Norfolk Island (e.g., Acizzia sp. A), but immatures have not been collected.

## ﻿Additional information

### Author ORCIDs

Francesco Martoni https://orcid.org/0000-0001-8064-4460

James M. H. Tweed https://orcid.org/0000-0002-5655-1499

Mark J. Blacket https://orcid.org/0000-0001-7864-5712

Diana M. Percy https://orcid.org/0000-0002-0468-2892

### Data availability

All of the data that support the findings of this study are available in the main text, or in public repositories as stated in the text. The data underpinning the analysis reported in this paper are deposited at GBIF, the Global Biodiversity Information Facility, and are available at https://doi.org/10.15468/sxpw8m.

## Supplementary materials

Supplementary material 1 Additional material collected and examined for this study Data type docx https://binary.pensoft.net/file/1333021This dataset is made available under the Open Database License (http://opendatacommons.org/licenses/odbl/1.0/). The Open Database License (ODbL) is a license agreement intended to allow users to freely share, modify, and use this Dataset while maintaining this same freedom for others, provided that the original source and author(s) are credited.Francesco Martoni, James M. H. Tweed, Mark J. Blacket, Diana M. Percy
