# Supplementary material for: ﻿An annotated checklist of the psyllids (Hemiptera, Psylloidea) of Norfolk Island with keys to species, new records, and descriptions of two new endemic species
Source: Zookeys. 2025 May 19;1238:297–348. doi: 10.3897/zookeys.1238.124535 (PMC12123370; doi:10.3897/zookeys.1238.124535)
Supplement: Supplementary material 1 — Additional material collected and examined for this study [file zookeys-1238-297_article-124535__-s001.docx]

**Supplementary Table 1:** Additional material collected and examined for this study.

| **Species** | **Collection Date** | **ID** | **Location** | **Males** | **Females** | **Nymphs** |
| --- | --- | --- | --- | --- | --- | --- |
| ***Blastopsylla occidentalis*** | 2022 | FM033 | **Norfolk Is.** National Park  Bullocks Hut Rd  13 Oct 2022 – F. Martoni  On *Eucalyptus botryoides* | 0 | 1 | 0 |
| ***Blastopsylla occidentalis*** | 2022 | FM036A | **Norfolk Is.** National Park  Anson Road  13 Oct 2022 – F. Martoni  On *Eucalyptus botryoides* | 25 | 19 | 0 |
| ***Blastopsylla occidentalis*** | 2022 | FM039 | **Norfolk Is.** N.P. Greg Quintal Rd  -29.019773, 167.927089  13 Oct 2022 – F. Martoni  On *Eucalyptus botryoides* | 4 | 12 | 0 |
| ***Blastopsylla occidentalis*** | 2022 | FM081A | **Norfolk Is.** Mill Rd  -29.05144,167.93928  17 Oct 2022 – F. Martoni  On *Eucalyptus* sp. | 11 | 13 | 3 |
| ***Blastopsylla occidentalis*** | 2022 | FM115C | **Norfolk Is.** Selwyn Pine Rd  -29.027318, 167.954960  18 Oct 2022 – F. Martoni  On *Eucalyptus* sp. | 46 | 64 | 0 |
| ***Blastopsylla occidentalis*** | 2022 | FM162 | **Norfolk Is.** Selwyn Pine Rd  -29.023191, 167.946495  21 Oct 2022 – F. Martoni  On *Eucalyptus* sp. | 1 | 3 | 0 |
| ***Blastopsylla occidentalis*** | 2022 | FM175 | **Norfolk Is.** Selwyn Pine Rd  -29.027318, 167.954960  22 Oct 2022 – F. Martoni  On *Eucalyptus* sp. | 129 | 117 | 2 |
| ***Blastopsylla occidentalis*** | 2013 | AW-12-97 | **Norfolk Is.** Selwyn Rd  11 Jul 2013 – A. Wells  On *Eucalyptus botryoides* | 2 | 4 | 0 |
| ***Blastopsylla occidentalis*** | 2013 | LAM5757 | **Norfolk Is.** National Park  10 Jul 2013 – L. Mound  On *Eucalyptus microcorys* | 3 | 3 | 0 |
| ***Blastopsylla occidentalis*** | 2012 | NI-2012-63 | **Norfolk Is.** National Park  22 Dec 2012 – L. Mound  On *Eucalyptus* cf. *botryoides* | 4 | 5 | 0 |
| ***Cardiaspina fiscella*** | 2022 | FM115B | **Norfolk Is.** Selwyn Pine Rd  -29.027318, 167.954960  18 Oct 2022 – F. Martoni  On *Eucalyptus* sp. | 15 | 3 | 0 |
| ***Cardiaspina fiscella*** | 2022 | FM162 | **Norfolk Is.** Selwyn Pine Rd  -29.023191, 167.946495  21 Oct 2022 – F. Martoni  On *Eucalyptus* sp. | 2 | 1 | 0 |
| ***Cardiaspina fiscella*** | 2022 | FM175 | **Norfolk Is.** Selwyn Pine Rd  -29.027318, 167.954960  22 Oct 2022 – F. Martoni  On *Eucalyptus* sp. | 42 | 11 | 0 |
| ***Cardiaspina fiscella*** | 2013 | AW-12-97 | **Norfolk Is.** Selwyn Rd  11 Jul 2013 – A. Wells  On *Eucalyptus botryoides* | 1 | 1 | 0 |
| ***Cryptoneossa triangula*** | 2022 | FM124 | **Norfolk Is.** Prince Phillip Dr  -29.018985, 167.961167  19 Oct 2022 – F. Martoni  On *Eucalyptus* sp. | 0 | 1 | 0 |
| ***Cryptoneossa triangula*** | 2022 | FM036B | **Norfolk Is.** National Park  Anson Road  13 Oct 2022 – F. Martoni  On *Eucalyptus botryoides* | 1 | 3 | 0 |
| ***Cryptoneossa triangula*** | 2022 | FM115D | **Norfolk Is.** Selwyn Pine Rd  -29.027318, 167.954960  18 Oct 2022 – F. Martoni  On *Eucalyptus* sp. | 1 | 0 | 0 |
| ***Glycaspis granulata*** | 2022 | FM115A | **Norfolk Is.** Selwyn Pine Rd  -29.027318, 167.954960  18 Oct 2022 – F. Martoni  On *Eucalyptus* sp. | 0 | 1 | 0 |
| ***Glycaspis granulata*** | 2022 | FM175 | **Norfolk Is.** Selwyn Pine Rd  -29.027318, 167.954960  22 Oct 2022 – F. Martoni  On *Eucalyptus* sp. | 0 | 1 | 0 |
| ***Glycaspis granulata*** | 2013 | AW-12-97 | **Norfolk Is.** Selwyn Rd  11 Jul 2013 – A. Wells  On *Eucalyptus botryoides* | 1 | 1 | 0 |
| ***Mesohomotoma hibisci*** | 2022 | FM041 | **Norfolk Is.** Anson Bay Car Park  -29.010591, 167.924713  14 Oct 2022 – F. Martoni  On *Hibiscus tiliaceus* | 15 | 4 | 16 |
| ***Mesohomotoma hibisci*** | 2022 | FM042 | **Norfolk Is.** Cook Memorial  -29.003007, 167.943700  14 Oct 2022 – F. Martoni  On *Hibiscus tiliaceus* | 2 | 1 | 18 |
| ***Mesohomotoma hibisci*** | 2022 | FM076 | **Norfolk Is.** Cook Memorial  -29.003007, 167.943700  16 Oct 2022 – F. Martoni  On *Hibiscus tiliaceus* | 0 | 0 | 29 |
| ***Mesohomotoma hibisci*** | 2022 | FM159 | **Norfolk Is.** Rocky Point Road  -29.05144,167.93928  20 Oct 2022 – F. Martoni  On *Hibiscus tiliaceus* | 8 | 4 | 0 |
| ***Mesohomotoma hibisci*** | 2013 | AW-002 | **Norfolk Is.** Airport  21 Oct 2013 – A. Wells  On *Hibiscus tiliaceus* | 2 | 7 | 2 |
| ***Mesohomotoma hibisci*** | 2013 | AW-12-83 | **Norfolk Is.** Taylor’s Rd  10 Jul 2013 – A. Wells  On *Dodonaea viscosa* | 0 | 2 | 0 |
| ***Mesohomotoma hibisci*** | 2012 | NI-2012-57 | **Norfolk Is.**  22 Dec 2012 – L. Mound | 1 | 3 | 0 |
| ***Mesohomotoma hibisci*** | 2012 | LAM5675 | **Norfolk Is.** Airport  21 Dec 2012 – L. Mound  On *Hibiscus tiliaceus* | 4 | 2 | 0 |
| ***Acizzia acaciaebaileyanae*** | 2023 | JT001 | **Norfolk Is.** Selwyn Pine Rd.  13 Mar 2023 – J.H. Tweed  On *Acacia podalyriifolia* | 34 | 77 | 11 |
| ***Acizzia acaciaebaileyanae*** | 2023 | JT002 | **Norfolk Is.** Lower J.E. Road  13 Mar 2023 – J.H. Tweed  On *Acacia podalyriifolia* | 0 | 7 | 3 |
| ***Acizzia acaciaebaileyanae*** | 2013 | AW-12-98 | **Norfolk Is.** Selwyn Rd  11 Jul 2013 – A. Wells  On *Acacia podalyriifolia* | 5 | 8 | 0 |
| ***Acizzia acaciaebaileyanae*** | 1922 | 010715752  [NHMUK] | **NEW ZEALAND**, Governors Bay  05 Aug 1922 – J.F. Tafley | 0 | 0 | 2 |
| ***Acizzia hakeae*** | 2023 | NI2025 | **Norfolk Is.** N.P, Site 20  -29.01769, 167.94414  Malaise Trap, 22 Oct-1 Nov 2023, JMH Tweed. | 0 | 1 | 0 |
| ***Acizzia* sp. A** | 2022 | FM001 | **Norfolk Is.** Rocky Point Rd  -29.05144,167.93928  20 Oct 2022 – F. Martoni  On *Dodonaea viscosa* | 0 | 1 | 0 |
| ***Acizzia* sp. B** | 2023 | JT003 | **Norfolk Is.** New Cascade Rd  11 Mar 2023 – J.H. Tweed  On *Acacia spirorbis* | 40 | 46 | 27 |
| ***Heteropsylla cubana*** | 2022 | FM163 | **Norfolk Is.** Selwyn Pine Rd  -29.023191, 167.946495  21 Oct 2022 – F. Martoni  On *Leucaena* sp. | 10 | 13 | 8 |
| ***Bactericera cockerelli*** | 2022 | FM129 | **Norfolk Is.** Bullocks Hut Rd  -29.00441,167.9271  16 Mar 2022 – F. Martoni  On *Solanum lycopersicum* | 2 | 7 | 0 |
| ***Bactericera cockerelli*** | 2013 | AW-031 | **Norfolk Is.** Hemus Rd  25 Oct 2013 – A. Wells  On *Solanum* sp. | 3 | 4 | 0 |
| ***Bactericera cockerelli*** | 2013 | LAM5839 | **Norfolk Is.** Hemus Rd  25 Oct 2013 – L. Mound  On *Capsicum* sp. | 0 | 3 | 0 |
| ***Powellia vitreoradiata*** | 2022 | FM002 | **Norfolk Is.** National Park  Mt. Pitt Track  19 Oct 2022 – F. Martoni  On *Pittosporum bracteolatum* | 2 | 5 | 0 |
| ***Powellia vitreoradiata*** | 2023 | FM003 | **Norfolk Is.** N.P.  Captain Cook Rd Gate  06 Nov 2023 – J.H. Tweed  On *Pittosporum undulatum* | 5 | 5 | 0 |
| ***Powellia vitreoradiata*** | 2023 | FM004 | **Norfolk Is.** N.P. Bridle Track  09 Oct 2023 – J.H. Tweed  Swept from ferns under *Pittosporum* sp. | 15 | 13 | 0 |
| ***Powellia vitreoradiata*** | 2013 | AW-018 | **Norfolk Is.** Tavener orchard  23 Oct 2013 – A. Wells  On *Pittosporum undulatum* | 1 | 9 | 0 |
| ***Powellia vitreoradiata*** | 2013 | LAM5823 | **Norfolk Is.** Red Rd  23 Oct 2013 – L. Mound  On *Pittosporum* sp. | 4 | 9 | 0 |
| ***Powellia vitreoradiata*** | 2013 | AW-12-95 | **Norfolk Is.** N.P. Palm Glen  11 Jul 2013 – A. Wells  On *Alyxia gynopogon* | 1 | 0 | 0 |
| ***Powellia vitreoradiata*** | 2013 | LAM5761 | **Norfolk Is.** N.P. Palm Glen  11 Jul 2013 – L. Mound  On *Pittosporum bracteolatum* | 2 | 2 | 0 |
| ***Powellia vitreoradiata*** | 2013 | AW-12-84 | **Norfolk Is.** Taylor’s Rd  10 Jul 2013 – A. Wells  On *Pittosporum undulatum* | 5 | 4 | 2 |
| ***Powellia vitreoradiata*** | 2013 | AW-12-70 | **Norfolk Is.** Highlands Ecolodge  08 Jul 2013 – A. Wells  On *Pittosporum bracteolatum* | 0 | 2 | 0 |
| ***Powellia vitreoradiata*** | 2012 | LAM5681 | **Norfolk Is.** Burnt Pine  22 Dec 2012 – L. Mound  On *Pittosporum undulatum* | 5 | 3 | 0 |
| ***Powellia vitreoradiata*** | 2024 | WN001 | **NEW ZEALAND**, MC, Halswell  43°34'26.2"S 172°34'27.2"E  24 Oct 2024 W. Nelson  On *Pittosporum* sp. | 0 | 0 | 10 |
| ***Powellia vitreoradiata*** | 1994 | 27052-94-1091  010719949  [NHMUK] | **UK**, Truro, Cornwall  28 Jun 1994 Mellor  On *Pittosporum* *tobira* | 0 | 0 | 4 |
